# Shifting Baselines: Longitudinal Reductions in EEG Beta Band Power Characterize Resting Brain Activity with Intensive Meditation

**DOI:** 10.1007/s12671-022-01974-9

**Published:** 2022-09-20

**Authors:** Alea C. Skwara, Brandon G. King, Anthony P. Zanesco, Clifford D. Saron

**Affiliations:** 1 Center for Mind and Brain, University of California, Davis, 267 Cousteau Place, Davis, CA 95616 USA; 2 Department of Psychology, University of Miami, Coral Gables, FL USA

**Keywords:** Meditation, EEG, Beta, Resting state, Domain generalization, State versus trait

## Abstract

**Objectives:**

A core assumption of meditation training is that cognitive capacities developed during formal practice will transfer to other contexts or activities as expertise develops over time. This implies that meditation training might influence domain-general neurocognitive systems, the spontaneous activity of which should be reflected in the dynamics of the resting brain. Previous research has demonstrated that 3 months of meditation training led to reductions in EEG beta band power during mindfulness of breathing practice. The current study extends these findings to ask whether concomitant shifts in power are observed during 2 min of eyes closed rest, when participants are not explicitly engaged in formal meditation.

**Methods:**

Experienced meditation practitioners were randomly assigned to practice 3 months of focused attention meditation in a residential retreat, or to serve as waitlist controls. The waitlist controls later completed their own 3-month retreat. Permutation-based cluster analysis of 88-channel resting EEG data was used to test for spectral changes in spontaneous brain activity over the course of the retreats.

**Results:**

Longitudinal reductions in EEG power in the beta frequency range were identified and replicated across the two independent training periods. Less robust reductions were also observed in the high alpha frequency range, and in individual peak alpha frequency. These changes closely mirror those previously observed during formal mindfulness of breathing meditation practice.

**Conclusions:**

These findings suggest that the neurocognitive effects of meditation training can extend beyond the bounds of formal practice, influencing the spontaneous activity of the resting brain. Rather than serving as an invariant baseline, resting states might carry meaningful training-related effects, blurring the line between state and trait change.

**Supplementary Information:**

The online version contains supplementary material available at 10.1007/s12671-022-01974-9.

A central claim of Buddhist contemplative traditions is that training in meditation can bring about lasting changes in the nature and habits of the mind (e.g., Dalai Lama & Cutler, 2009; Wallace 2006). Consistent with these claims, different forms and regimens of meditation training have been shown to influence capacities as diverse as attentional stability (e.g., Lutz et al., 2009; van Leeuwen et al., 2012; Zanesco et al., 2013, 2019), stress buffering (e.g., Creswell & Lindsay, 2014), emotion regulation and reactivity (e.g., Lutz et al., 2008; Rosenberg et al., 2015), and prosociality (e.g., Ashar et al., 2016; Condon et al., 2013; Weng et al., 2017). Critically, these changes may extend well beyond the bounds of formal meditation sessions, influencing broad domains of daily life (e.g., Donald et al., 2019; Sahdra et al., 2011; Skwara et al., 2017). The manifestation of these effects across domains that are not explicitly trained implies that meditation training might alter domain-general neurocognitive systems. Generalized changes in such systems should theoretically be observed across a variety of situations, contexts, and, notably, in the spontaneous neural activity of the brain at rest (e.g., Bauer et al., 2019).

The brain is remarkably responsive to changes in environment and behavior. For instance, immobilizing a person’s arm for only 48 h can lead to neuroplastic changes in functional brain connectivity (Newbold et al., 2020). The capacity of the brain to undergo reorganization has also been observed in the context of contemplative practice. Experienced meditators show persistent shifts in functional (e.g., Davidson & Lutz, 2008; Hasenkamp & Barsalou, 2012) and structural (e.g., Fox et al., 2014; Lumma et al., 2018) brain organization. These changes can be observed during active meditation practice (e.g., Braboszcz et al., 2017; Fucci et al., 2018; Lee et al., 2018; Saggar et al., 2012), during task engagement (e.g., Desbordes et al., 2012; van Leeuwen et al., 2012; Zanesco et al., 2019), and in the functional architecture of the resting brain (e.g., Dentico et al., 2018; Hasenkamp & Barsalou, 2012; Zanesco et al., 2021).

Much of the neuroscientific literature on meditation has focused on investigations of brain activity during formal meditation practice (for reviews, see Cahn & Polich, 2006; Lee et al., 2018; Lomas et al., 2015). During formal meditation practice, practitioners engage with a specified set of mental activities for a given period of time. These formal sessions are typically undertaken in a particular physical posture, such as sitting or lying down; and are conditioned by social, ethical, and other contextual factors (Lutz et al., 2015). For example, during mindfulness of breathing meditation, a practitioner might sit quietly in an upright posture, focusing on the sensations of breath at the aperture of their nostrils or the rising and falling of their abdomen. When they notice that their mind has wandered, they are instructed to gently redirect it back to the breath (Gunaratana, 2002). Through repeated practice, practitioners cultivate the ability to regulate attention and to volitionally maintain awareness on a chosen object. Over time, improvements in the ability to direct and sustain attention are thought to extend beyond the meditative context and generalize to other activities (Dalai Lama & Cutler, 2009; Lutz et al., 2015; Wallace, 2006).

While the boundary conditions of formal meditation sessions are often clearly delineated, the *effects* of meditation training are much less circumscribed (Cahn & Polich, 2006; Skwara et al., 2017). Experiential (e.g., Dalai Lama & Cutler, 2009; Kabat-Zinn, 2013) as well as empirical (e.g., Desbordes et al., 2012; Fox et al., 2014; Hasenkamp & Barsalou, 2012) accounts suggest that neurocognitive changes instantiated through meditation extend beyond the bounds of formal practice, and the brain systems and cognitive mechanisms engaged through various meditative practices are implicated in a wide array of psychological processes (Dahl et al., 2015; Lutz et al., 2015). As such, meditation-related changes in neurocognitive systems may manifest across a range of different contexts and outcomes. Experientially, shifts in perception and awareness experienced during formal practice may, over time, extend into daily life in ways that are both pervasive and persistent (Dalai Lama & Cutler, 2009; Davidson & Kaszniak, 2015; Kabat-Zinn 2013; Wallace, 2006), blurring the line between meditative states and everyday experience.

Evidence for meditation-related domain generalization can be gleaned from tasks that engage the specific skills or capacities ostensibly trained by a given meditation practice, as well as from tasks that cut across cognitive and affective domains that are not specifically targeted by that practice. For example, previous investigations of a waitlist-controlled longitudinal study of focused attention meditation—known as the Shamatha Project—found that 3 months of intensive training in a retreat environment lead to improvements in the capacity to regulate one’s attention (MacLean et al., 2010; Sahdra et al., 2011; Shields et al., 2020; Zanesco et al., 2016, 2018, 2019), but also to alterations in emotional responses to suffering (Rosenberg et al., 2015), and improvements socioemotional functioning (Sahdra et al., 2011). It remains unclear, however, what brain processes support the tendency for cognitive capacities engaged during formal practice to generalize across diverse psychological domains.

To the extent that meditation training leads to generalized changes in cognition and behavior, there should be observable shifts in the activity of underlying brain systems that support these functions. One method for quantifying the functioning of such brain systems is to examine neural oscillations, as indexed by electrical activity at the scalp (e.g., Buzsaki et al., 2012). In an analysis of EEG recorded during mindfulness of breathing meditation as part the Shamatha Project, replicable changes in brain activity were also observed (Saggar et al., 2012). Participants who received meditation training demonstrated significant reductions in band power in the beta frequency range, as well as reductions in peak individual alpha frequency. These reductions replicated across two independent retreat interventions, and were not observed in waitlist controls. Building on research implicating beta band activity in attentional orienting to sensory information (e.g., Pfurtscheller & Lopes da Silva, 1999; Schubert et al., 2009; van Ede et al., 2011), these findings were interpreted to reflect enhanced attention to, and sensory processing of, the subtle sensations of breath during mindfulness of breathing developed through intensive practice (Saggar et al., 2012).

The present report leverages data from the Shamatha Project to ask whether the neuroelectric changes observed during formal meditation practice might generalize to an uninstructed resting state. We hypothesized that 3 months of residential training would alter brain oscillatory activity during quiet rest. We further hypothesized that these changes would mirror those previously observed during mindfulness of breathing meditation, namely, overall reductions in beta band power and individual alpha frequency. By extending this investigation to the resting brain, we hoped to shed light on neurocognitive factors that might support generalized changes in meditation-related processes over a period of intensive practice.

## Method

### Participants

We recruited experienced meditation practitioners through advertisements in print and online Buddhist publications. Following recruitment, 60 eligible participants (32 females, 28 males; *M*_age_ = 48 years, range = 22 to 69) were randomly assigned to an initial training group (*n* = 30) or a waitlist control group (*n* = 30) using a stratified matching procedure. The groups were matched at baseline on age, sex, ethnicity, and major personality characteristics, as well as several cognitive task variables assessed prior to assignment (for details of recruitment and group matching, see MacLean et al., 2010; Shields et al., 2020). They were also matched on lifetime meditation experience, with an overall mean of 2610 cumulative hours (initial training: *M* = 2549 h, range = 250 to 9500; waitlist control: *M* = 2,668, range = 250 to 15,000). In addition, participants were screened for medical conditions and Axis I psychiatric diagnoses as assessed by the Mini International Neuropsychiatric Interview screen (Sheehan et al., 1998) and a clinical interview administered by a licensed clinical psychologist.

One waitlist participant left the study after completing the control assessments due to circumstances unrelated to the study. This left a total of 29 participants for the second training intervention.

### Procedures

The waitlist design included two 3-month-long residential meditation retreats conducted in the spring and fall of 2007. The two retreats were formally identical in training structure and were held in the same scenic retreat environment. During the first retreat (Retreat 1), active training participants lived and practiced meditation on-site at Shambhala Mountain Center in Red Feather Lakes, CO. Waitlist control participants continued with their daily lives during this time and were flown to the retreat center to complete on-site assessments at the beginning, middle, and end of the intervention period. At each assessment, waitlist control participants arrived at the retreat center approximately 3 days (range = 65–75 h) prior to their laboratory session for an initial acclimatization period to adjust to the altitude (~2500 m) and natural environment. Following Retreat 1, waitlist control participants returned to the retreat center and underwent their own 3-month retreat intervention as active training participants (Retreat 2). Thus, the design comprised three participant statuses: Retreat 1 active training participants, Retreat 1 waitlist controls, and Retreat 2 active training participants. The Retreat 2 training participants were the same participants as Retreat 1 waitlist controls and completed their first assessment as active training participants approximately 3 months after their final assessment as waitlist controls.

While on retreat, training participants practiced meditation for 6 to 8 h a day, under the guidance of Dr. B. Alan Wallace, an experienced Buddhist teacher and contemplative scholar. Participants gathered twice daily to engage in guided group meditation and instruction and met individually with Dr. Wallace once a week. The meditation instructions were drawn from the Theravada and Mahayana Buddhist traditions and included Shamatha and four immeasurables practices (described in Wallace, 2006). Shamatha techniques aim to develop stability of attention, perceptual vividness, and concentration, and were the primary practices taught on retreat. These consisted of (1) *mindfulness of breathing*, in which attention is focused on the sensations of the breath; (2) *observing mental events*, in which attention is turned to all forms of mental phenomena; and (3) *observing the nature of consciousness*, in which focus is placed on the awareness of being aware. The four immeasurables of *loving-kindness*, *compassion*, *empathetic joy*, and *equanimity* aim to cultivate beneficial aspirations for the self and others (for a description, see Rosenberg et al., 2015; Wallace, 2010). The four immeasurables were taught as supportive practices, and participants reported in engaging in four immeasurables practice for approximately 45 min per day, on average. Overall, training participants reported devoting most of their practice time to mindfulness of breathing (for full practice time details, see Sahdra et al., 2011).

### Measures

On-site laboratory assessments were conducted at the beginning (pre-assessment), middle (mid-assessment), and end (post-assessment) of each retreat. At each assessment, participants completed approximately 4 h of testing on each of two consecutive days. The results of these assessments can be found in several other reports (e.g., MacLean et al., 2010; Rosenberg et al., 2015; Saggar et al., 2012; Sahdra et al. 2011; Shields et al., 2020; Zanesco et al., 2019). All testing took place in two field laboratories with darkened, sound-attenuated testing and control rooms built on-site at the retreat center. Retreat 1 training participants completed a total of three on-site assessments, while waitlist controls completed a total of six assessments—three as controls in Retreat 1, and three as active training participants in Retreat 2.

#### Resting EEG

Resting EEG was collected as the first laboratory task at each assessment. Continuous EEG was recorded across 4 min of rest, divided into four 1-min segments of eyes open and eyes closed rest (open, closed, closed, open). At the beginning of each resting segment, participants were instructed via an audio recording: “For the next sixty seconds please sit quietly with your eyes closed [open].” Importantly, however, participants’ interpretation of these instructions may have also been influenced by prior instructions given during the EEG set-up prior to the period of quiet rest. Before the resting period, the instructions “Rest without engaging in any particular form of directed mental activity” were displayed to participants on screen during set-up. Because our goal was to investigate brain activity in the absence of an explicit task, these instructions were intentionally non-directive and avoided any mention of meditation or mind wandering.

In our prior report, most participants practiced mindfulness of breathing with their eyes closed. Therefore, for consistency with these data, we included data from the eyes closed resting epochs only. In addition, only participants who had usable EEG data at all assessment points of a given retreat were included in the analyses (in total, four files were excluded upon initial inspection; eight more were excluded following preprocessing). This resulted in a total of 52 participants (27 female; *M*_age_ = 48.16 years, *SD*_age_ = 14.18, range = 22.25 to 69.69) providing a total of 156 observations for Retreat 1. Of these, 25 were training participants (12 female; age: *M*_age_ = 49.70, *SD*_age_ = 12.88, range = 23.90 to 69.69), and 27 were waitlist controls (15 female; age: *M*_age_ = 46.74, *SD*_age_ = 15.39, range = 22.25 to 65.16). For Retreat 2, 26 training participants (13 female; *M*_age_ = 46.47 years, *SD*_age_ *=* 15.56, range = 22.25 to 65.16) provided 78 observations.

##### Data Acquisition and Processing

EEG was recorded with the BioSemi ActiveTwo system (http://www.biosemi.com) at a sampling rate of 2048 Hz. Easycap electrode caps (http://www.easycap.de) were fitted with BioSemi electrode holders in an 88-channel equidistant montage, and individual electrode locations were localized using a Polhemus Patriot digitizer (http://www.polmehus.com). On participant request, some electrodes (primarily at frontopolar locations) were not inserted or were removed to minimize discomfort. The EEG recordings were band-pass filtered offline between 0.1 and 200 Hz (zero-phase; roll-off; 12 dB/octave LP, 24 dB/octave HP) and then referenced to the average of all remaining channels. Data preprocessing was conducted in BESA 5.2 (www.besa.de). Channels with very low signal quality were discarded prior to analysis, and data were manually marked to remove extreme artifacts and intermittent high amplitude EMG contamination.

##### Separating Neural from Non-neural Signal Sources

Following the process outlined in Saggar et al. (2012), second-order blind source identification (SOBI; Belouchrani et al., 1997) was used to separate signals of putative neural origin from non-neural sources. SOBI is a method similar to ICA that functions to separate signal components. Unlike ICA, which examines only momentary correlations, SOBI uses joint-diagonalization of correlation matrices at multiple temporal delays. This is used to identify maximally independent sources by minimizing the sum of the squared cross-correlations of all pairs of sources across all temporal delays. We used 41 temporal delays, *τ* = [1:1:10, 12:2:20, 25:5:100, 120:20:300] ms, as recommended in Tang et al. (2005). The two consecutive 1-min segments of eyes closed resting EEG were concatenated and submitted to SOBI. A novel SeMi-automatic Artifact Removal Tool (SMART; https://stanford.edu/~saggar/Software.html; Saggar et al., 2012) was used to generate signal source topography, spectra, autocorrelation, and time series for inspection. These SMART outputs were used to manually classify signal sources as neural or non-neural (e.g., EMG, ocular artifacts, line noise) in origin (see Saggar et al., 2012, for examples of SMART output and a discussion of the parameters considered in source classification).

##### Reconstruction and Conversion into Standardized Electrode Space

Following application of SOBI, sources identified as non-neural were removed and the remaining putative neural sources were reconstructed into the original 88-channel montage. To ensure that channel locations were standardized across participants, the reconstructed montage was then transformed into a standard 81-channel montage (international 10-10 system) using spherical spline interpolation (smoothing factor of 2e-07) as implemented in BESA 5.2. Eight channels of the standard 81-channel montage (AF9, Fpz, Fp2, Nz, AF10, CB1, CB2) did not have corresponding nearest electrode sites in the original montage and so were removed from the interpolated locations, yielding a final standardized 73-channel montage.

##### Scalp Current Density

We used the MATLAB CSD Toolbox (http://psychophysiology.cpmc.columbia.edu/Software/CSDtoolbox; Kayser & Tenke, 2006), to transform data from the standardized 73-channel montage into a reference-free estimation of scalp current source density (CSD) using spherical spline interpolation (Perrin et al., 1989). Resulting CSD units are given in μV/m^2^. The surface Laplacian was estimated as the second derivative of the scalp potential and smoothed by a lambda factor of 2e-05. Transformation of scalp voltage to CSD minimizes the effects of volume conduction and improves visualization of scalp topographic differences (Kayser & Tenke, 2012, 2015).

##### Power Spectral Estimation

The 2 min of reconstructed EEG data were segmented into 2-s (4096 point) segments with 50% overlap. Power spectra estimates, averaged over 2-s windows, were computed in the MATLAB FieldTrip package (Oostenveld et al., 2011) using multi-tapered power spectral density estimation (Mitra & Pesaran, 1999; Oostenveld et al., 2011) and a Hanning window (Welch, 1967) at 0.5 Hz resolution. Frequency bands were defined relative to each individual’s peak alpha frequency (IAF). IAF was calculated within a frequency range of 7 Hz (*f*_1_) to 14 Hz (*f*_2_) using the center-of-gravity method of Klimesch (1999):

$${\alpha}_{IAF}=\frac{\Sigma_{i={f}_1}^{f_2}\left(a\left({f}_i\right)\times {f}_i\right)}{\Sigma_{i={f}_1}^{f_2}a\left({f}_i\right)}$$

where *f*_*i*_ denotes the power-spectral estimate at frequency *i*. For each EEG recording, IAF values were calculated for each channel separately, and then averaged across all channels to obtain a single mean estimate of IAF per participant per assessment. Frequency bands were then calculated for each participant at each assessment based on their mean IAF. Table 1 presents the IAF frequency band definitions and resultant IAF-based frequency band ranges used in the current data set, alongside the canonical frequency band definitions. After the IAF-based frequency ranges were defined, power was estimated within each band by averaging over the 2 min of eyes closed EEG for each individual’s idiosyncratic frequency range. The results presented below are based on a single estimate of power (μV^2^/m^2^) per frequency band for each electrode, participant, and assessment. We additionally conducted all analyses using canonical fixed frequency bands. Using fixed bands did not result in changes to our core findings. These results are presented in the Supplementary Information.

**Table 1:** IAF-based Frequency Band Values and Ranges

Frequency Band	Range Based on IAF	Range in Current Data Set	Fixed Band Range
Delta	1.0 – 0.4 × IAF Hz	1.0 – 3.93 (0.19) Hz	0.1 – 4 Hz
Theta	0.4 × IAF – 0.6 × IAF Hz	3.93 (0.19) – 5.90 (0.29) Hz	4 – 8 Hz
Alpha	0.6 × IAF – 1.2 × IAF Hz	5.90 (0.29) – 11.80 (0.58) Hz	8 – 13 Hz
*Alpha 1*	*0.6 × IAF – 0.8 × IAF Hz*	*5.90 (0.29) – 7.86 (0.39) Hz*	*----*
*Alpha 2*	*0.8 × IAF – IAF Hz*	*7.87 (0.39) – 9.83 (0.48) Hz*	*----*
*Alpha 3*	*IAF – 1.2 × IAF Hz*	*9.83 (0.48) – 11.80 (0.58) Hz*	*----*
Beta	1.2 *×* IAF – 30 Hz	11.80 (0.58) – 30 Hz	13 – 30 Hz
Gamma	30 – 50 Hz	30 – 50 Hz	30 – 50 Hz
IAF	---	8.69 – 11.28 Hz^†^	---

Ranges for the current data set are presented as the mean (SD) of the lower and upper limits of each IAF-based frequency band across all participants and assessments. Canonical band definitions are as described in Cohen (2014), with the exception of alpha sub-bands, for which there are no established canonical ranges independent of IAF. ^†^Range for IAF is the absolute minimum and maximum observed in the current data set

#### Self-Reported Meditation Practice

While on retreat, participants recorded the amount of time, in minutes, that they had dedicated to meditation practice each day (see Sahdra et al., 2011). For each participant, we averaged these daily estimates across days to compute an index of average daily practice time. Prior to group assignment, participants provided self-reported histories of their past meditation practice. These reports were used to calculate estimated lifetime hours (see MacLean et al., 2010).

### Data Analyses

#### Non-parametric Permutation-Based Cluster Identification

We examined changes in electrode-wise band power estimates as a function of assessment (pre-, mid-, and post-retreat) using non-parametric cluster-based permutation testing, implemented with the *ft_freqstatistics* function in FieldTrip (Oostenveld et al., 2011). This data-driven approach identifies contiguous clusters of electrodes that demonstrate reliable changes in band power, while also controlling for multiple comparisons (Maris & Oostenveld, 2007). It is important to note that identified clusters provide evidence for differences between conditions—in this case across the three assessment points—but do not provide evidence for changes at any specific electrode site (see Sassenhagen & Draschkow, 2019, for the spatial limitations of cluster-based permutation tests).

A separate non-parametric permutation test was conducted for each participant status (Retreat 1 training, Retreat 1 control, and Retreat 2 training) and IAF-based frequency band (delta, theta, alpha, beta, gamma). In cases where change was identified in the alpha band, we conducted follow-up tests for changes in three alpha sub-bands (alpha 1, alpha 2, alpha 3), based on prior evidence for functional differences between lower (alpha 1, alpha 2) and upper (alpha 3) alpha (Klimesch, 1999).

First, for each electrode, change in band power across assessments was evaluated as a multivariate *F*-statistic, and electrodes demonstrating a significance level of *α* ≤ 0.05 were selected as candidate cluster members. These candidate electrodes were then grouped into clusters based on spatial adjacency. Cluster criteria were set such that each candidate electrode was required to have two adjacent electrodes that were also cluster candidates, resulting in a minimum cluster size of three electrodes. Cluster-level statistics were then calculated by taking the sum of the *F*-statistics of all electrodes comprising a cluster. The significance of this cluster statistic was assessed non-parametrically through 10,000 permutations of a Monte Carlo approximation. Finally, we subjected the resultant cluster probabilities to the false discovery rate (FDR) procedure of Benjamini and Hochberg (1995) to control for multiple comparisons. Cluster statistics that survive this correction indicate change across assessments that are larger than would be expected by chance.

#### Parametric Analysis: Mixed Models

Cluster-wise power estimates were subjected to parametric statistical testing to assess the significance and directionality of change across assessments as a function of participant status. First, band power at each electrode included in a significant cluster was log-transformed. Then, these values were averaged within each cluster to create a cluster-wise estimate of band power, or cluster mean, reported in log (μV^2^/m^2^). This was done for each individual at each assessment. Following the approach in Saggar et al. (2012), when a cluster was identified for a given participant status (e.g., Retreat 1 training), cluster mean power estimates based on the comprising electrodes were also calculated for the relevant comparison status. For example, if an alpha band cluster of 10 electrodes was identified in Retreat 1 training participants, a cluster mean of these 10 electrodes would also be generated for each Retreat 1 control participant. Likewise, if a cluster was identified for Retreat 2 training participants, this cluster was also applied to the data from these same participants as Retreat 1 waitlist controls. This allowed for direct parametric comparison of power change in corresponding sets of electrodes across experimental conditions.

Changes in cluster mean power were analyzed using linear mixed effects models implemented in SAS PROC MIXED version 9.4. Assessment (pre-, mid-, post-) and participant status (training, control) were included as fixed effects. In Retreat 1, status functioned as a between-groups effect (Retreat 1 training compared to Retreat 1 controls), while in Retreat 2 it served as a within-subjects contrast (Retreat 2 training participants compared to their prior status as Retreat 1 controls). A random effect of participant was included to allow for repeated measures within subjects. Parameters were estimated using restricted maximum likelihood, and degrees of freedom were calculated based on the Satterthwaite approximation.

Of primary interest was the assessment by status interaction, the presence of which would indicate that participants on retreat demonstrated a pattern of change across assessments that differed from participants not currently on retreat. This was followed by a test of the effect of assessment within each status and directed comparisons of model estimated marginal means. The effect of assessment was centered to pre-assessment and participant status was centered to the control group for all follow-up tests.

Changes in IAF were examined using an identical analytic procedure to that used in the parametric analysis of cluster mean power.

#### Associations Between Cluster Mean Power and Meditation Practice Hours

Correlations were computed between cluster mean power and the meditation practice variables of meditation practice hours while on retreat, and pre-assignment lifetime meditation experience (also in hours). For these correlations, we examined training participants only, collapsing across the two retreats to maximize statistical power. We examined associations between meditation practice variables and beta cluster mean power at the pre- and post-assessments, and change in power across the retreat intervention.

Additionally, we tested correlations between resting cluster mean power and cluster mean power in EEG collected during 6 min of mindfulness of breathing meditation recorded at each assessment as previously reported in Saggar et al. (2012).

Changes were quantified as difference scores from the pre-retreat to the post-retreat assessment, where negative scores indicate a reduction over the course of retreat. All variables were tested for normality using the Shapiro-Wilk test. We also checked for outliers (defined according to Tukey’s rule as 1.5 times the interquartile range below the first quartile, or above the third quartile). Associations between normally distributed variables were computed using Pearson correlation coefficients, calculated both with and without outliers. Associations including non-normally distributed variables were calculated using Kendall’s Tau. Because Kendall’s Tau is robust to outliers, these correlations were always conducted on all available data. Significance values were FDR adjusted using the Benjamini-Hochberg (1995) procedure.

## Results

### Retreat 1 Spectral Analysis

We first examined changes in IAF over the course of Retreat 1. Table 2 presents mean IAF for Retreat 1 training and control participants at each assessment. We observed a main effect of assessment, *F*(2, 100) = 9.75, *p* < .001, but no main effect of status, *F*(1, 50) = 0.18, *p* = .670, or interaction between assessment and status, *F*(2, 100) = 1.48, *p* = .234, indicating that the groups did not significantly differ in their change across time.

**Table 2:** Descriptive Statistics for Retreat 1 Dependent Measures

		Band Power			Meditation Practice Hours	
IAF		*Alpha Cluster*	*Alpha 3 Cluster*	*Beta Cluster*	*Daily Retreat*	*Lifetime*
Training Group					6.58 (1.36)	2408.00 (2684.23)
Pre	9.93 (0.36)	2.91 (0.93)	2.69 (0.95)	1.73 (0.63)		
Mid	9.81 (0.44)	2.78 (0.82)	2.49 (0.78)	1.50 (0.60)		
Post	9.79 (0.43)	2.59 (0.85)	2.34 (0.82)	1.40 (0.60)		
Waitlist Controls					--	2615.41 (3146.96)
Pre	9.93 (0.47)	3.53 (0.95)	3.29 (0.98)	2.18 (0.64)		
Mid	9.87 (0.48)	3.46 (0.86)	3.20 (0.85)	2.16 (0.60)		
Post	9.88 (0.54)	3.49 (0.75)	3.22 (0.79)	2.13 (0.49)		

Values are presented as mean (SD). Band power units are log (μV^2^/m^2^). Cluster means for waitlist controls (*n* = 27) are based on the clusters identified in Retreat 1 training participants (*n* = 25). Daily retreat hours represent the average time participants reported dedicating to practice in their daily logs while on retreat, and lifetime hours represent an estimate of lifetime practice hours at pre-assignment

In a previous report we observed retreat-related changes in IAF among this same participant cohort during mindfulness of breathing practice (Saggar et al., 2012). Therefore, as a follow-up to those findings, we chose to further explore changes in IAF during rest within each group in Retreat 1. A test of simple effects indicated that IAF significantly changed over assessments in training participants, *F*(2, 100) = 8.91, *p* < .001, but not in waitlist controls, *F*(2, 100) = 2.06, *p* = .133. Follow-up comparisons of model estimated means indicated that IAF did not significantly differ between training and control participants at the pre-retreat assessment (*b* = −0.01, *SE* = 0.13, *p* = .957, 95% CI [−0.26, 0.25]). Additional comparisons indicated that IAF in training participants decreased significantly from pre- to mid-retreat (*b* = −0.12, *SE* = 0.03, *p* < .001, 95% CI [−0.19, −0.05]), and from pre- to post-retreat (*b* = −0.14, *SE* = 0.04, *p* < .001, 95% CI [−0.21, −0.07]), but not from mid- to post-retreat (*b* = −0.01, *SE* = 0.04, *p* = .593, 95% CI [−0.08, 0.06]). However, there were no significant differences in IAF between the retreat and control groups at the mid-retreat (*b* = −0.06, *SE* = 0.13, *p* = .617, 95% CI [−0.32, 0.19]), or post-retreat (*b* = −0.09, *SE* = 0.13, *p* = .488, 95% CI [−0.34, 0.17]) assessments.

#### Cluster Identification

**Fig. 1:**
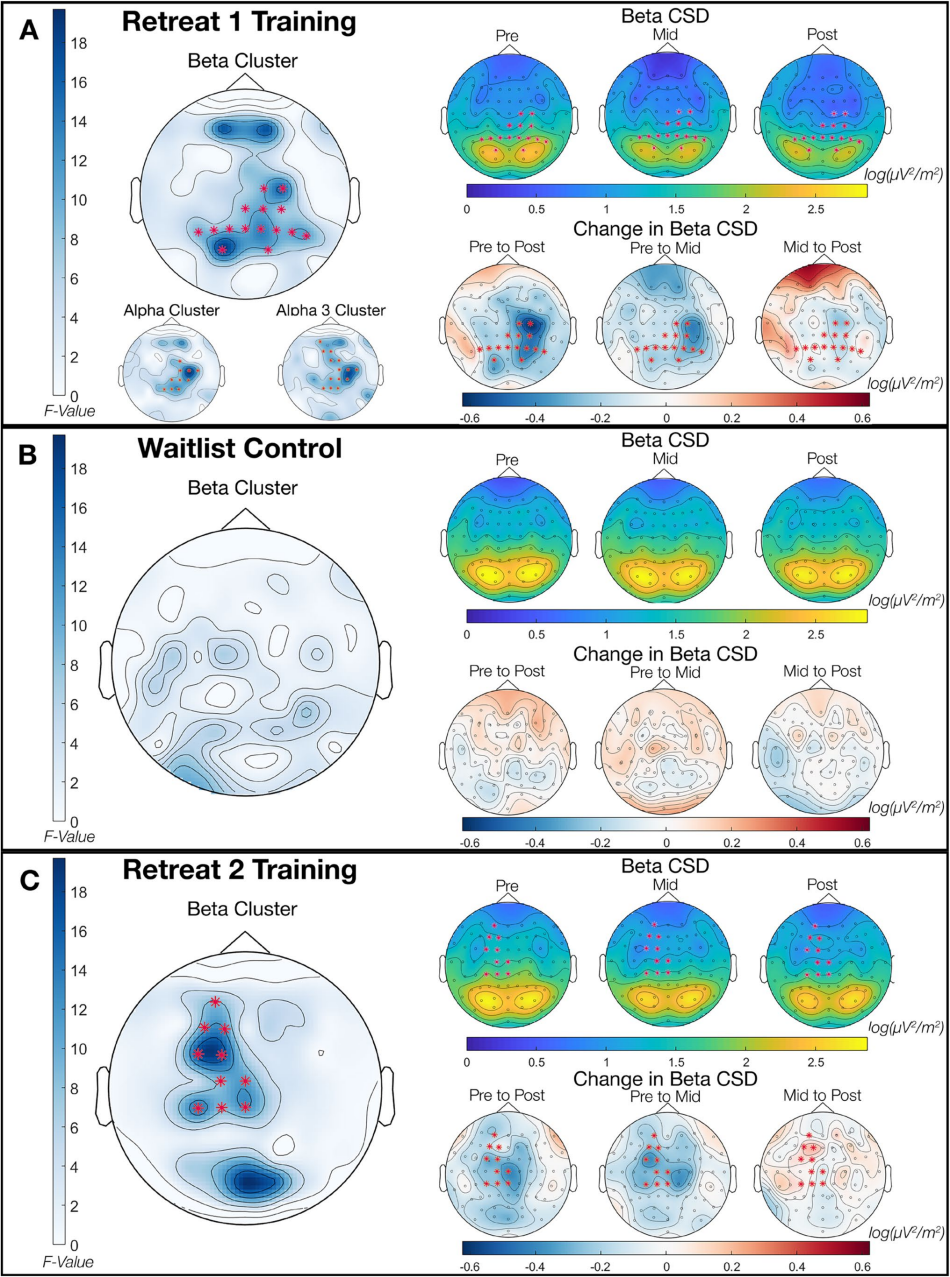
Identified clusters of current source density (CSD) power change across retreat in (**A**) Retreat 1 training participants, *n* = 25; (**B**) Retreat 1 waitlist controls, *n* = 27; and (**C**) Retreat 2 training participants (previously waitlist controls), *n* = 26. The asterisk (*) indicates electrodes that comprise a significant cluster. All cluster *p*s < 0.01. For each panel, the leftmost maps depict cluster statistic *F*-values; the right upper maps depict CSD beta power at pre-, mid-, and post-retreat; and the right lower maps depict raw subtracted differences in CSD power between assessments (e.g., “Pre to Post” = post – pre)

The non-parametric permutation analysis for Retreat 1 training participants indicated a significant change in alpha band power, *cluster statistic* = 108.99, *p* = .010, and beta band power, *cluster statistic* = 161.24, *p* = .004, across assessments (see Fig. 1A). We followed up on the identified alpha cluster in retreat participants by testing for clusters in alpha sub-bands. This analysis indicated significant band power differences between assessments in the upper alpha range only (i.e., alpha 3; IAF – 1.2 × IAF Hz), *cluster statistic* = 157.60, *p* = .003. An additional cluster was identified in alpha 2 that did not reach statistical significance, *cluster statistic* = 33.03, *p* = .060. No changes were indicated for the remaining bands in training participants. No clusters were identified in any band in waitlist controls.

**Fig. 2:**
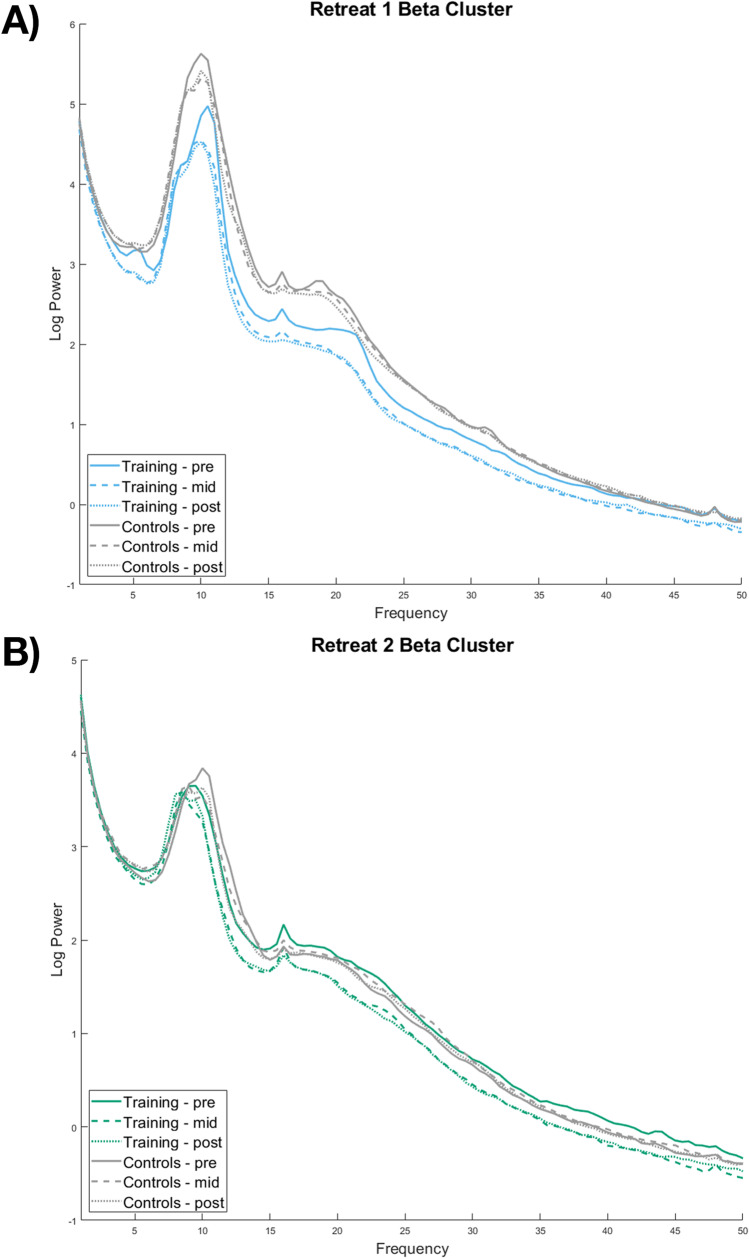
Mean power spectra (μV^2^/^m2^) in electrodes comprising the beta clusters identified in (**A**) Retreat 1 training participants (*n* = 25) and (**B**) Retreat 2 training participants (*n* = 26). Mean spectra for waitlist controls (*n* = 27) were also calculated for the comprising electrodes and are displayed next to training participants for comparison. Note that panel **B** therefore displays a comparison between waitlist participants as controls in Retreat 1 and as training participants in Retreat 2. Also note that the peak at 16 Hz is a known artifact due to the excitation frequency of the electrodermal measurement in the Biosemi system used for recording and does not reflect neural activity

Table 2 presents descriptive statistics for cluster mean power estimates (derived from identified clusters) for frequency bands demonstrating significant change across assessments. Estimates are given for training participants, in whom the clusters were identified, as well as the corresponding values for these clusters in waitlist controls. The findings of the cluster analysis for bands showing significant change (beta, whole band alpha, and the alpha 3 sub-band) in Retreat 1 training participants are shown in the left side of Fig. 1, panel A. The electrodes comprising identified clusters are superimposed as stars on the electrode-wise *F*-values. The leftmost column of panel B shows the *F*-values in the beta band for Retreat 1 controls. As no clusters were identified in this group, no electrodes are marked. Fig. 2, panel A, displays the average power from 1 to 50 Hz at each assessment averaged across the electrodes comprising the beta cluster identified in Retreat 1. This spectral visualization shows power decreases specific to the training group, with regions of exaggerated change extending across a broad range of frequencies corresponding to the beta band, as well as in a narrower frequency range under the alpha peak. Corresponding power spectra of the alpha and alpha 3 clusters can be found in Supplementary Materials.

#### Parametric Tests

**Fig. 3:**
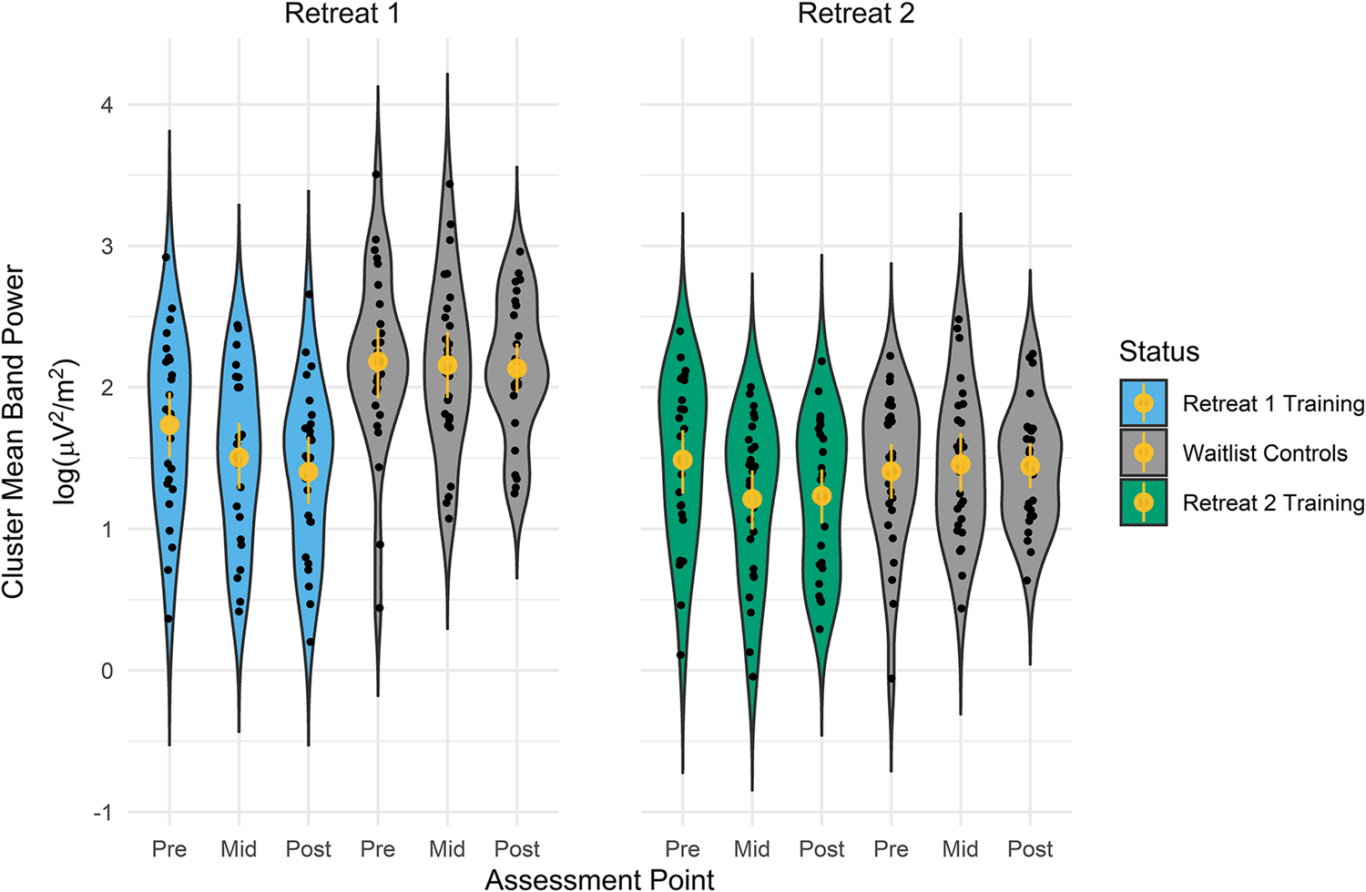
Beta cluster mean power at each assessment point in clusters identified for each retreat training group (Retreat 1, Retreat 2). Significant clusters were identified in the training groups only, and were then applied to the waitlist control group, for whom no significant clusters were identified. Thus, the two sets of data displayed for waitlist controls represent their cluster means for the electrodes comprising the beta clusters identified in Retreat 1 and Retreat 2 training participants, respectively. Black dots are individual data points, yellow circles are group means, and yellow lines are the standard error of the mean. Retreat 1, *n* = 25, Waitlist Control, *n* = 27. Retreat 2, *n* = 26. Note that the Waitlist Control and Retreat 2 Training groups represent the same participants before attending, and while attending retreat, respectively

We next examined condition differences in mean power for the identified alpha and beta band clusters (Fig. 3). As no clusters of significant change were identified in waitlist controls, their Retreat 1 cluster mean power estimates are based on the clusters identified for Retreat 1 training participants, which were applied to both groups (see “Method”).

For the alpha band cluster, type 3 tests of fixed effects indicated a main effect of assessment, *F*(2, 100) = 4.32, *p* = .002, a main effect of participant status *F*(1, 50) = 10.27, *p* = .002, and a non-significant interaction between assessment and status, *F*(2, 100) = 2.82, *p* = .065. Due to the lack of a significant interaction effect, no follow-up tests were conducted. For the cluster specific to upper alpha (alpha 3), there was a main effect of assessment, *F*(2, 100) = 6.08, *p* = .003, a main effect of status, *F*(1, 50) = 10.05, *p* = .003, and, again, a non-significant interaction between assessment and status, *F*(2, 100) = 2.81, *p* = .065.

For the beta band, there were significant main effects of assessment, *F*(2, 100) = 8.21, *p* < .001, and status, *F*(1, 50) = 15.29, *p* < .001, and a significant interaction between assessment and status, *F*(2, 100) = 4.64, *p* = .012. A test of simple effects within each group revealed a significant effect of assessment in training participants, *F*(2, 100) = 12.12, *p* < .001, but not in controls, *F*(2, 100) = 0.28, *p* = .760. Follow-up comparisons indicated that training and control participants significantly differed in cluster mean beta power at the pre-retreat assessment (*b* = −0.45, *SE* = 0.17, *p* = .001, 95% CI [−0.78, −0.12]), such that training participants had lower cluster mean beta power at the beginning of retreat. In addition, within-group comparisons indicated that training participants decreased significantly in cluster mean beta band power from pre- to mid-retreat (*b* = −0.23, *SE* = 0.07, *p* = .001, 95% CI [−0.36, −0.09]), and from pre- to post-retreat (*b* = −0.33, *SE* = 0.07, *p* < .001, 95% CI [−0.46, −0.19]), but not from mid- to post-retreat (*b* = −0.10, *SE* = 0.07, *p* = .153, 95% CI [−0.23, 0.04]). Consistent with these patterns, training participants demonstrated significantly lower cluster mean beta band power than did waitlist controls at the mid-retreat (*b* = −0.65, *SE* = 0.17, *p* < .001, 95% CI [−0.98, −0.32]) and post-retreat (*b* = −0.73, *SE* = 0.17, *p* < .001, 95% CI [−1.06, −0.40]) assessments.

### Retreat 2 Spectral Analysis

For analyses of Retreat 2 data, we compared active training participants to their own prior status as Retreat 1 waitlist controls.

We first checked for change in IAF across assessments. Mean values for IAF in Retreat 2 participants are presented in Table 3. For IAF, there was a significant main effect of assessment, *F*(2, 124) = 16.57, *p* < .001, a significant main effect of participant status, *F*(1, 125) = 55.78, *p* < .001, and a significant interaction between assessment and status, *F*(2, 124) = 6.81, *p* = .002. A test of simple effects further indicated a significant effect of assessment when participants were on retreat, *F*(2, 124) = 21.70, *p* < .001, but not when they served as waitlist controls, *F*(2, 124) = 1.30, *p* = .277. Follow-up comparisons revealed no difference in pre-assessment IAF as a function of participant status (*b* = −0.08, *SE* = 0.04, *p* = .082, 95% CI [−0.17, 0.01]). Moreover, during Retreat 2, IAF significantly decreased from pre- to mid-assessment (*b* = −0.21, *SE* = 0.04, *p* < .001, 95% CI [−0.29, −0.12]), and from pre- to post-retreat (*b* = −0.28, *SE* = 0.04, *p* < .001, 95% CI [−0.36, −0.19]), but not from mid- to post-retreat (*b* = −0.07, *SE* = 0.04, *p* = .120, 95% CI [−0.15, 0.02]). Consistent with these patterns, Retreat 2 participants demonstrated significantly lower IAF during training than as waitlist controls, at both the mid-retreat (*b* = −0.22, *SE* = 0.04, *p* < .001, 95% CI [−0.31, −0.13]) and post-retreat (*b* = −0.30, *SE* = 0.04, *p* < .001, 95% CI [−0.39, −0.21]) assessments.

**Table 3:** Descriptive Statistics for Retreat 2 Dependent Measures

		Band Power	Meditation Practice Hours	
**IAF**		*Beta cluster*	*Daily Retreat*	*Lifetime*
In Training			6.26 (1.54)	2688.46 (3315.96)
Pre	9.84 (0.50)	1.49 (0.59)		
Mid	9.63 (0.50)	1.21 (0.57)		
Post	9.56 (0.49)	1.24 (0.53)		
As Controls			--	2615.41 (3146.96)
Pre	9.93 (0.47)	1.41 (0.53)		
Mid	9.87 (0.48)	1.46 (0.54)		
Post	9.88 (0.54)	1.44 (0.42)		

Values are presented as means (SD). Band power units are log(μV^2^/m^2^). Cluster means for participants as controls (*n* = 27) are based on the clusters identified in these participants during Retreat 2 training (*n* = 26). Daily retreat hours represent the average time participants reported dedicating to practice in their daily logs while on retreat, and lifetime hours represent an estimate of lifetime practice hours at pre-assignment. Note that lifetime hours vary slightly between the “in training” and “as controls” conditions as slightly different subsets of participants in this group provided usable data in each of these conditions

#### Cluster Identification

We next conducted non-parametric cluster analyses of Retreat 2 training participants across their 3 assessments while on retreat. Non-parametric tests indicated a significant difference in beta band power, *cluster statistic* = 121.53, *p* = .008. No clusters were identified in any other band. The identified cluster can be seen in the left side of Fig. 1, panel C, and descriptive statistics for cluster mean power estimates can be found in Table 3. Figure 2, panel B, shows the average power from 1 to 50 Hz at each assessment in the electrodes comprising the significant cluster identified in Retreat 2. This visualization of the cluster spectra demonstrates power reductions specific to the training condition, with exaggerated change in frequencies corresponding to the beta range and potentially extending into higher frequencies.

#### Parametric Tests

No clusters were identified in Retreat 2 training participants when they served as waitlist controls during Retreat 1 (see “Retreat 1 Spectral Analysis”). Consequently, all Retreat 2 cluster mean power estimates are based on the significant cluster identified in Retreat 2 training participants, applied to their EEG collected during each of the two retreats. Using these estimates, we tested for differences in cluster mean power in Retreat 2 participants in their status as active retreat participants versus waitlist controls (Fig. 3). Tests of fixed effects for mean beta power indicated no main effect of assessment, *F*(2, 124) = 2.50, *p* = .087, a significant main effect of status, *F*(1, 128) = 7.65, *p* = .007, and a significant interaction between assessment and status, *F*(2, 124) = 4.82, *p* = .010. A test of simple effects revealed a significant effect of assessment when Retreat 2 participants were actively on retreat, *F*(2, 124) = 6.99, *p* = .001, but not when they served as controls, *F*(2, 124) = 0.20, *p* = .820. Follow-up comparisons further indicated that cluster mean beta band power did not differ at the pre-retreat assessment as a function of participant status (*b* = 0.07, *SE* = 0.08, *p* = .421, 95% CI [−0.10, 0.23]). During Retreat 2, participants demonstrated a significant decrease in cluster mean beta band power from pre- to mid-retreat (*b* = −0.27, *SE* = 0.08, *p* = .001, 95% CI [−0.44, −0.11]), and from pre- to post-retreat (*b* = −0.25, *SE* = 0.08, *p* = .002, 95% CI [−0.41, −0.09]), but not from mid- to post-retreat (*b* = 0.02, *SE* = 0.08, *p* = .780, 95% CI [−0.14, 0.18]). Consistent with these patterns, Retreat 2 participants demonstrated significantly lower cluster mean beta band power at the mid-retreat (*b* = −0.26, *SE* = 0.08, *p* < .001, 95% CI [−0.42, −0.09]) and post-retreat (*b* = −0.22, *SE* = 0.08, *p* = .008, 95% CI [−0.39, −0.06]) assessments than they did as waitlist controls.

### Associations Between Cluster Mean Power and Other Measures

#### Correlations with Meditation Practice

We examined the relationship between cluster mean beta power during rest and the meditation practice metrics of estimated lifetime meditation hours and average reported daily meditation hours while on retreat. As both measures of practice hours were shown violate the assumptions of normality (daily hours: *W* = 0.94, *p* = .009; lifetime hours: *W* = 0.67, *p* < .001), Kendall’s Tau was used. Lifetime hours showed a significant positive correlation with cluster mean beta power at the beginning, *r*_*τ*_ = .27, *p*_ajd_ = .039, and end, *r*_*τ*_ = .24, *p*_ajd_ = .047, of retreat, such that participants with greater meditation experience coming into retreat tended to have higher beta power, when both variables were converted to rank order. There was no significant relationship between lifetime hours and change in cluster mean beta power while on retreat, *r*_*τ*_ = −.05, *p*_ajd_ = .760. Similarly, there were no significant relationships between average daily meditation practice hours while on retreat and cluster mean beta power, all *ps*_ajd_ >.720.

#### Correlations with Beta Band Reductions During Mindfulness of Breathing

**Fig. 4:**
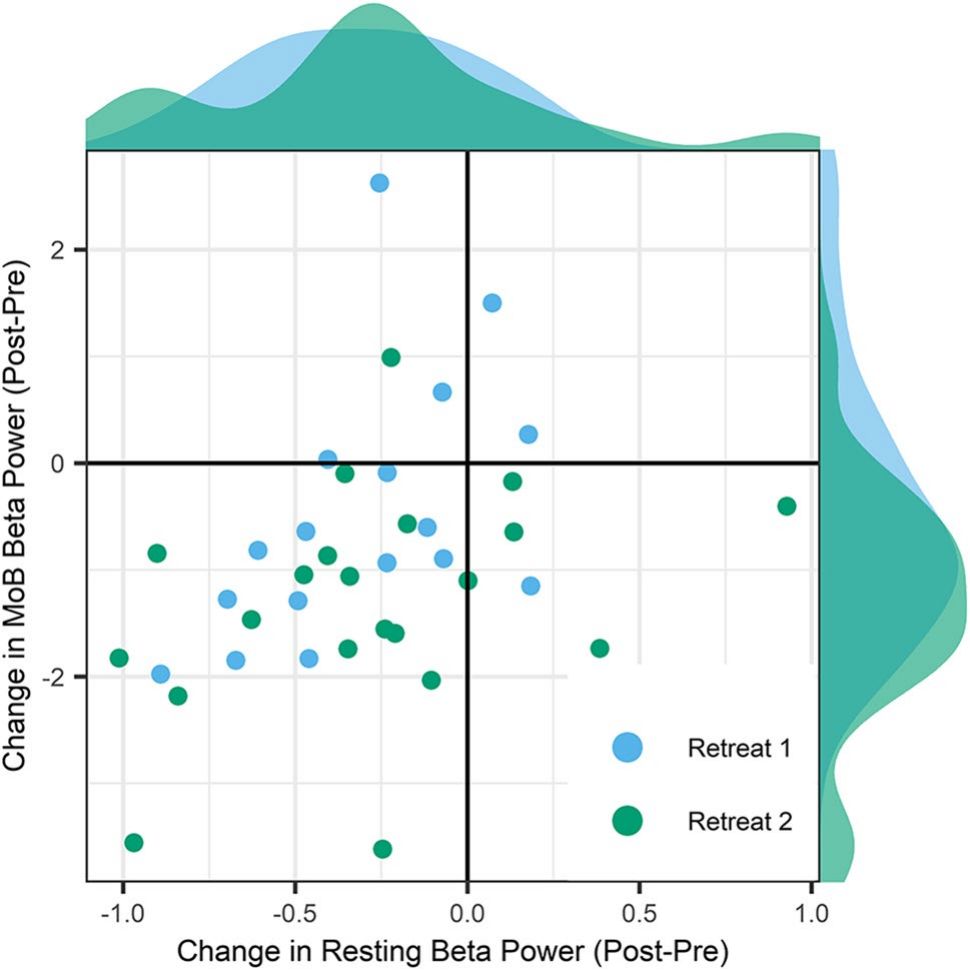
Correlation between change in beta band cluster mean power during eyes closed rest and during mindfulness of breathing practice in active retreat participants, *r*(36) = .37, *p*_adj_ = .02, 95% CI [0.06, 0.62] with all data included; *r*(34) = .43, *p* = .009, 95% CI [.12, .67] with outliers removed. The density distribution of each variable is represented on the respective axis

Finally, we calculated Pearson correlations between cluster mean beta band power during eyes closed rest and corresponding values from a previous analysis of mindfulness of breathing practice in these same participants (see Saggar et al., 2012 for a full description of the clusters identified in that analysis). Beta band cluster mean power was strongly correlated between rest and mindfulness of breathing in retreat participants at the pre-, *r*(36) = .61, *p*_adj_ < .001, 95% CI [.36, .78], mid-, *r*(36) = .55, *p*_adj_ < .001, 95% CI [.28, .74], and post-retreat, *r*(36) = .62, *p*_adj_ < .001, 95% CI [.38, .79] assessments. There was also a moderate correlation between changes in resting cluster mean power and changes in mindfulness of breathing, *r*(36) = .37, *p*_adj_ = .02, 95% CI [.06, .62]. This association is shown in Fig. 4. There were no outliers in cluster mean beta power at rest or during mindfulness of breathing at any assessment. There was one outlier for change in cluster mean beta power during rest and one outlier during mindfulness of breathing. After removing these outliers, the correlation between these measures remained significant, *r*(34) = .43, *p* = .009, 95% CI [.12, .67], indicating that the relationship was not driven by extreme values.

## Discussion

This study was motivated by a central question in contemplative research: can engaging in dedicated periods of meditation practice lead to generalized changes outside of formal practice? To this end, we examined changes in the spontaneous activity of the brain over the course of intensive meditation training. We had participants engage in focused attention (*shamatha*) meditation practice for 6 to 8 h a day and measured continuous EEG activity during a period of uninstructed rest. We found power reductions in the high alpha and beta bands, as well as reductions in IAF, during an eyes closed resting task over the course two 3-month-long retreat interventions. Importantly, the reductions in beta band activity replicated across two independent training periods, mirroring longitudinal changes we previously observed in EEG collected during active practice of mindfulness of breathing meditation in these same participants (Saggar et al., 2012). By contrast, changes in alpha were identified in only one of the two retreat groups. Our findings demonstrate that intensive meditation training can result in neurophysiological changes that extend beyond the bounds of formal practice.

Our findings, as well those of Saggar et al. (2012), were seemingly specific to longitudinal changes in the beta band. While the identified reductions in IAF suggest change in the peak frequency of the EEG signal—which could, in turn, affect band power by shifting the range of IAF-defined bands—an analysis of fixed frequency bands indicated that IAF shifts did not underlie the observed reductions in beta band power (see Supplementary Materials). The consistency of these effects across meditation and rest points to beta band activity as a potential indicator of domain-general change in neural processes resulting from this type of meditation training.

Beta band activity is broadly implicated in a range of neurocognitive functions and network dynamics, including sensorimotor processing (e.g., Pfurtscheller & Lopes da Silva, 1999; van Ede et al., 2011), cognitive effort (Kopell et al., 2010), attentional orienting (van Ede et al., 2011), top-down control of visual attention (Bastos et al., 2015; Buschman & Miller, 2007), predictive coding of the sensory environment (e.g., Arnal & Giraud, 2012), and working memory (Axmacher et al., 2008; Miller et al., 2018). Recent work also demonstrates the relevance of beta band activity to cross-domain inhibitory control. For instance, Castiglione et al. (2019) showed that actively preventing a thought from coming to mind elicits increases in beta power similar to those elicited when stopping a physical action. These findings offer support for the idea that beta power may reflect the activity of neurocognitive networks that exert their effects across different modalities.

One possible interpretation of the current findings is that power reductions at rest signal alterations in the structure, efficiency, or dynamics of the default mode or other large-scale brain networks (e.g., de Pasquale et al., 2012; Wens et al., 2019). Power in the beta range during rest appears to fluctuate with BOLD activity in several canonical resting state networks, notably showing a positive correlation with activity in regions of the default mode network, and a negative correlation with those of the dorsal attention network (Mantini et al., 2007). Beta band activity is also associated with functional connectivity between and within resting state networks (de Pasquale et al., 2012, 2018; Wens et al., 2019), with band-limited power in the beta frequency corresponding to moments of high network efficiency (Betti et al., 2021). Thus, it appears that beta activity may relate to efficiency of communication between the brain’s core networks (Betti et al., 2021).

Consistent with this, research in experienced meditators indicates that long-term meditation training may lead to altered resting functional connectivity and reduced activity within the default mode network (Berkovich-Ohana et al., 2014; Brewer et al., 2011; Garrison et al., 2015). Moreover, in our own work in the Shamatha Project, we found retreat-related changes in dynamic patterns of resting EEG microstates (Zanesco et al., 2021). These lines of research suggest that the observed reductions in beta over retreat could be reflective of altered patterns of functional connectivity, and possibly changes in the predominance of default mode activity during uninstructed rest (e.g., Bauer et al., 2019). This implies that—rather than being specific to meditative states—the observed retreat-related changes in beta band activity could indicate broad shifts in baseline patterns of brain activity and its underlying functional architecture.

While other studies have characterized meditation-related reductions in the beta frequency range *during* meditation practice compared to rest (during Shamatha practice: Saggar et al., 2012; during Zen practice: Faber et al., 2015, Hauswald et al., 2015; see also Cahn & Polich, 2006 and Lomas et al., 2015, for reviews), to our knowledge, the only other study to identify changes in the beta frequency range in the resting brains of experienced meditators found *increases* in power following a day of Vipassana or Metta practice (Dentico et al., 2018). Interestingly, in the current study, we found that greater lifetime meditation experience before entering retreat was associated with higher overall beta power. However, neither previous lifetime experience nor practice time while on retreat were related to observed power reductions over retreat. This suggests that the current findings may reflect the holistic training experience of retreat, or that the acute effects of intensive meditation might differ from the cumulative, lasting effects of lifelong practice. That our between-individual and within-individual effects were in opposite directions speaks to the complex trajectories of meditation-related change, particularly in the context of longer term or intensive training (see, for example, King et al., 2019), and points to the importance of mapping within person variability in addition to group-level differences.

A number of recent studies have found that different aspects of meditation practice and experience may be indexed by different components of the EEG signal. For example, a study by DeLosAngeles et al. (2016) found that increased alpha band power characterized focused attention meditation when compared to rest, but that decreasing beta band power was associated with self-reported depth of meditation during practice. Similarly, Bauer et al. (2019) found a reduction in activity and functional connectivity in the default mode network of experienced meditators at rest compared to novices, but comparative increases in these same metrics during focused attention meditation. Of particular relevance to the current study, Rodriguez-Larios et al. (2021) found that modulations in individualized alpha as well as power in the alpha/beta range associated with meditation and mind wandering differed between experienced meditators and novices. In their study, experienced meditators showed decreases in IAF and power in the alpha/beta range, as well as a steeper 1/f slope, during meditation compared to rest—patterns not observed in novices. In contrast, novices demonstrated increased alpha/beta power during episodes of mind wandering while actively engaged in meditation practice—an effect not noted in experienced meditators. These findings indicate alterations to both oscillatory and non-oscillatory aspects of the EEG signal (Donoghue et al., 2022) as a function of meditation experience, as well as more broadband changes spanning multiple frequency bands.

Similarly, our current findings were not entirely restricted to the beta band. In Retreat 1, we identified a cluster of change in the alpha range, which an analysis of alpha sub-bands localized to high alpha (alpha 3). Power reductions in the alpha range did not replicate in Retreat 2 and were not robust enough to reach significance in between-group parametric analyses. However, the presence of these clusters indicates that spectral changes extended beyond the beta range. Potential broadband spectral change was further suggested by visualizations of the full power spectra obtained from the significantly identified beta clusters (shown in Fig. 2). In line with our cluster analyses, clear reductions were apparent for training participants in the beta range. However, reductions also appeared to manifest across a broader frequency spectrum. Consistent with this, a broadband cluster analysis—not restricted by frequency bands—found a significant cluster of change extending across a wide range of frequencies in Retreat 2 (see Supplementary Materials). Moreover, global visualizations of the strength of electrode-wise change in both retreats were similarly suggestive of broadband reductions, with elevated power change in the alpha, and particularly the beta, ranges (Supplementary Figs. 2 and 3).

Whether these broader changes represent a distinct phenomenon from the reductions in beta power is an open question. Power in the beta (Ploner et al., 2006; Tamura et al., 2005) and high alpha bands (Klimesch, 1999; Samaha et al., 2017) share a degree of functional overlap: both appear to be inversely related to cortical excitability, such that lower power is associated with greater activation of local cortical networks. Suppression in these frequencies may reflect disinhibition of underlying neural assemblies, allowing for greater cortical excitability and thus enhanced stimulus processing. This interpretation is consistent with reports of reduced acoustic startle habituation among experienced practitioners of Tibetan non-dual traditions (i.e., Dzogchen or Mahamudra; Antonova et al., 2015), suggesting that the sensory systems of experienced meditators may maintain their responsiveness in contexts that would typically induce habituation. However, it is also possible that the observed alpha and beta clusters relate to distinct functional changes not sufficiently captured in the current study, or that apparent frequency-specific changes were an artifact of our band-based analytic approach (e.g., Donoghue et al., 2022). Future work is needed to delineate the spectral specificity of meditation-related changes in trait-like neural patterning.

The discrepancies between the aforementioned findings in the literature could result from various methodological sources, including (a) differing cognitive-affective processes engaged across distinct styles of practice (e.g., Dahl et al., 2015; Lutz et al., 2015), (b) design and analytic approaches—including the choice of comparison groups (e.g., novice or experienced meditators) and baseline conditions (e.g., instructed mind-wandering, uninstructed rest; see Cahn & Polich, 2006; Davidson & Kaszniak, 2015; Van Dam et al., 2018), and the methodology used to characterize neural oscillations (e.g., Donoghue et al., 2022; Rodriguez-Larios et al., 2021), and (c) the experience levels of practitioner groups, who may display unique trajectories of training-related change (e.g., King et al., 2019; Skwara et al., 2017). Indeed, the effects of meditation practice and training may manifest differently as a function of these design decisions, pointing to the important, perhaps even deterministic, role that these choices play in outcomes of meditation studies (Van Dam et al., 2018).

### Similarities Between Rest and Mindfulness of Breathing

The retreat-related changes in brain activity observed in the current study mirror those previously identified in these same participants during active practice of mindfulness of breathing (Saggar et al., 2012). Both analyses found reductions in frontoparietal EEG power in the beta band, as well as IAF slowing. The similarity of these findings raises questions regarding the meaning of ostensible state versus trait measures in the context of intensive meditation training.

First, might our participants have been meditating when asked to rest quietly? While our instructions discouraged participants from engaging in active, formal meditation practice during the resting period, we were intentionally non-directive as to what mind-state participants *should* maintain. In contrast to more explicit resting instructions given in other studies (e.g., instructed mind wandering; see Braboszcz et al., 2017; Cahn et al., 2010), our instructions allowed us to observe more naturalistic changes in the resting brain, albeit while sacrificing a degree of methodological control and certainty. We therefore cannot fully rule out the possibility that participants were engaging in meditation practice during the resting period. Nevertheless, participants were instructed to avoid “engaging in any particular form of directed mental activity.” In addition, we included a separate guided meditation task at each assessment that was distinct from the resting task, of which participants were aware. Therefore, we believe it unlikely that most participants were intentionally and actively engaging in a formal meditation practice.

The second question pertains to the fluidity of meditative versus non-meditative states for experienced practitioners, and what it means to “rest” in the context of retreat. The observed reductions in beta power during rest were strongly correlated with previously reported reductions in beta during mindfulness of breathing at each assessment, while retreat-related changes in the two measures were moderately correlated. This lends support to the idea that at least a portion of the observed reductions in beta power reflect patterns of change common to quiet rest and formal practice. While formal meditation practice is undertaken within relatively circumscribed bounds, the effects of training may be more far-reaching, leading to pervasive shifts in perception, emotion, and cognition that have long been reported in traditional practitioner accounts (e.g., Dalai Lama & Cutler, 2009; Wallace 2006). From this perspective, meditation is not discontinuous with other domains of experience. This may especially be true in the context of a meditation retreat, where participants are encouraged to imbue all their daily activities with contemplative awareness. Thus, one’s baseline quality of awareness may, over time, come to more closely resemble those states cultivated during sessions of formal meditation. This points to the complexity of separating state and trait effects, and lends support to dimensional, process-oriented models of meditation-related change (e.g., Dahl et al., 2015; Lutz et al., 2015). Taken in the broader context of other findings from the Shamatha Project (e.g., Rosenberg et al., 2015; Sahdra et al., 2011; Shields et al., 2020; Zanesco et al., 2018), the present work speaks to the wide range of domains that were affected by the same retreat experience. Importantly, the current findings demonstrate that rest is not an invariant baseline and that it may be altered in meaningful ways through meditative practice.

### Limitations and Future Research

Our study is limited by having a waitlist, rather than active, control group. Additionally, all of our participants were experienced meditators. As such, our findings speak to patterns that occur during an intensive period of retreat training in already-experienced practitioners, and may not pertain to changes that occur earlier in the developmental trajectory of contemplative practice or in non-intensive interventions (e.g., King et al., 2019). Though participants dedicated many of their waking hours to formal meditation practice during retreat, our findings might also reflect the complex and non-specific influences of retreat experience—including diet, distance from the stressors and commitments of daily life, social and spiritual support, and the idyllic natural setting of the retreat center—rather than the effects of a specific meditative practice in isolation (King et al., 2019).

The lack of a direct statistical comparison to EEG during mindfulness of breathing practice limits our ability to interpret the relative strength and similarity of observed changes to those of active meditation. To clarify the relationship between changes in the functional architecture of the resting brain and brain activity during meditative practice, future longitudinal studies should characterize within-individual trajectories of change in each of these conditions separately, as well as in direct comparison to one another. Based on the current findings, we expect that similar patterns of change will be reflected in both conditions, with the stronger instantiations during active practice.

Additionally, while other work in this participant cohort has linked brain activity at rest to self-reported felt qualities of awareness (Zanesco et al., 2021), the current findings do not provide a direct experiential or behavioral link. To test the functional relevance of meditation-related changes in resting brain dynamics, future studies should attempt to relate resting neurophysiology to behavioral measures that tap skills cultivated during active meditation practice. We expect that training-related shifts in resting brain activity should predict concomitant improvements in relevant behavioral performance.

Finally, methodological issues complicate our interpretation of neural oscillatory and frequency-specific effects (see Donoghue et al., 2022). We used IAF-based bands to account for individual variation in peak alpha frequency and visualized the spectra within identified clusters to visually confirm the presence of peaks in spectral power. However, we did not apply formal peak detection methods in the current analysis (e.g., Donoghue et al., 2020; Kosciessa et al., 2020; Watrous et al., 2018). As such, despite the apparent specificity of the current findings, it is possible that the results were at least partially driven by changes in aperiodic signal components. Future studies should employ emerging methods that decompose the EEG signal into putative periodic and aperiodic components (Donoghue et al., 2020, 2022). Such parameterization of the power spectrum can provide greater certainty as to the origin of observed shifts meditation-related neural activity. Following on the current findings and the observations of Rodriguez-Larios and colleagues (2021), we predict that approaches incorporating parameterization of the power spectrum will reveal training-related changes in both periodic and aperiodic components of the EEG signal.

## Supplementary Information

Supplementary Fig. 1 (PNG 641 kb)

High resolution image (TIF 5569 kb)

Supplementary Fig. 2 (PNG 965 kb)

High resolution image (TIF 5830 kb)

Supplementary Fig. 3 (PNG 979 kb)

High resolution image (TIF 5810 kb)

Supplementary Fig. 4 (PNG 477 kb)

High resolution image (TIF 10685 kb)

Supplementary Fig. 5 (PNG 895 kb)

High resolution image (TIF 152 kb)

(DOCX 18.8 kb)

## Data Availability

The data from this study are freely available in the Open Science Framework: https://osf.io/xjfyv/?view_only=87168d639a42440996fc20f4ae2b90cc
