# Supplementary figures and images for: Shifting Baselines: Longitudinal Reductions in EEG Beta Band Power Characterize Resting Brain Activity with Intensive Meditation
Source: Mindfulness (N Y). 2022 Sep 20;13(10):2488–506. doi: 10.1007/s12671-022-01974-9 (PMC9568471; doi:10.1007/s12671-022-01974-9)

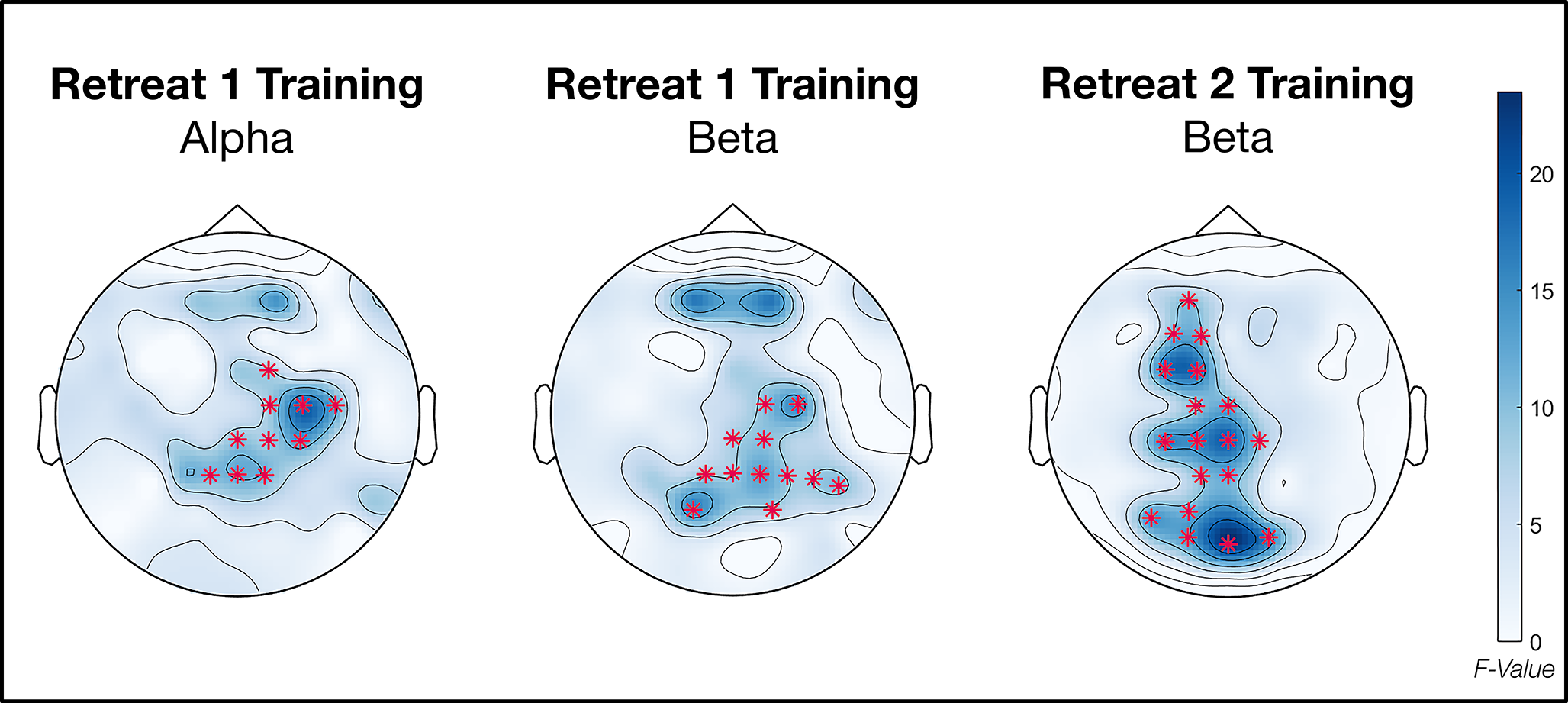

Supplement: Supplementary file 1 — (PNG 641 kb) [file 12671_2022_1974_Fig5_ESM.png]

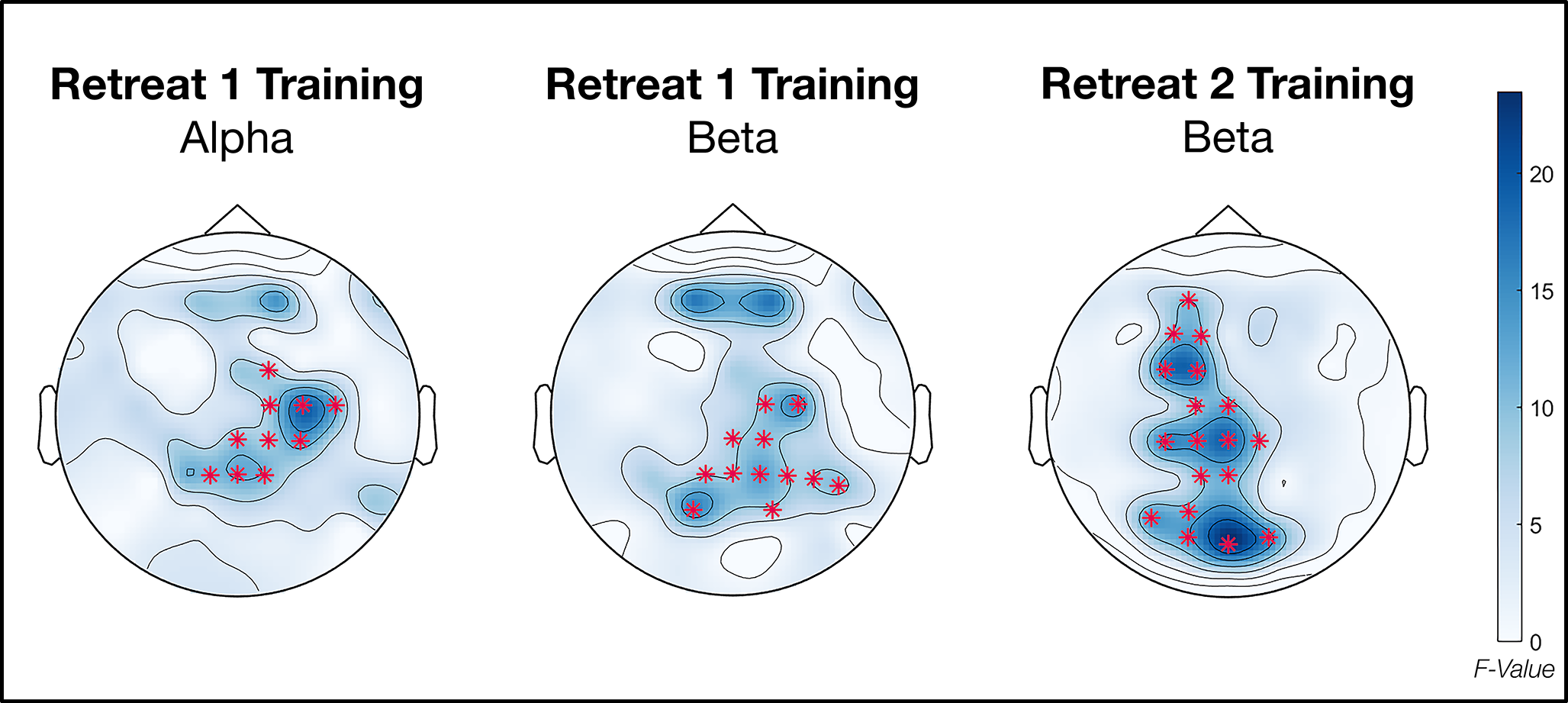

Supplement: Supplementary file 2 — High resolution image (TIF 5569 kb) [file 12671_2022_1974_MOESM1_ESM.tif]

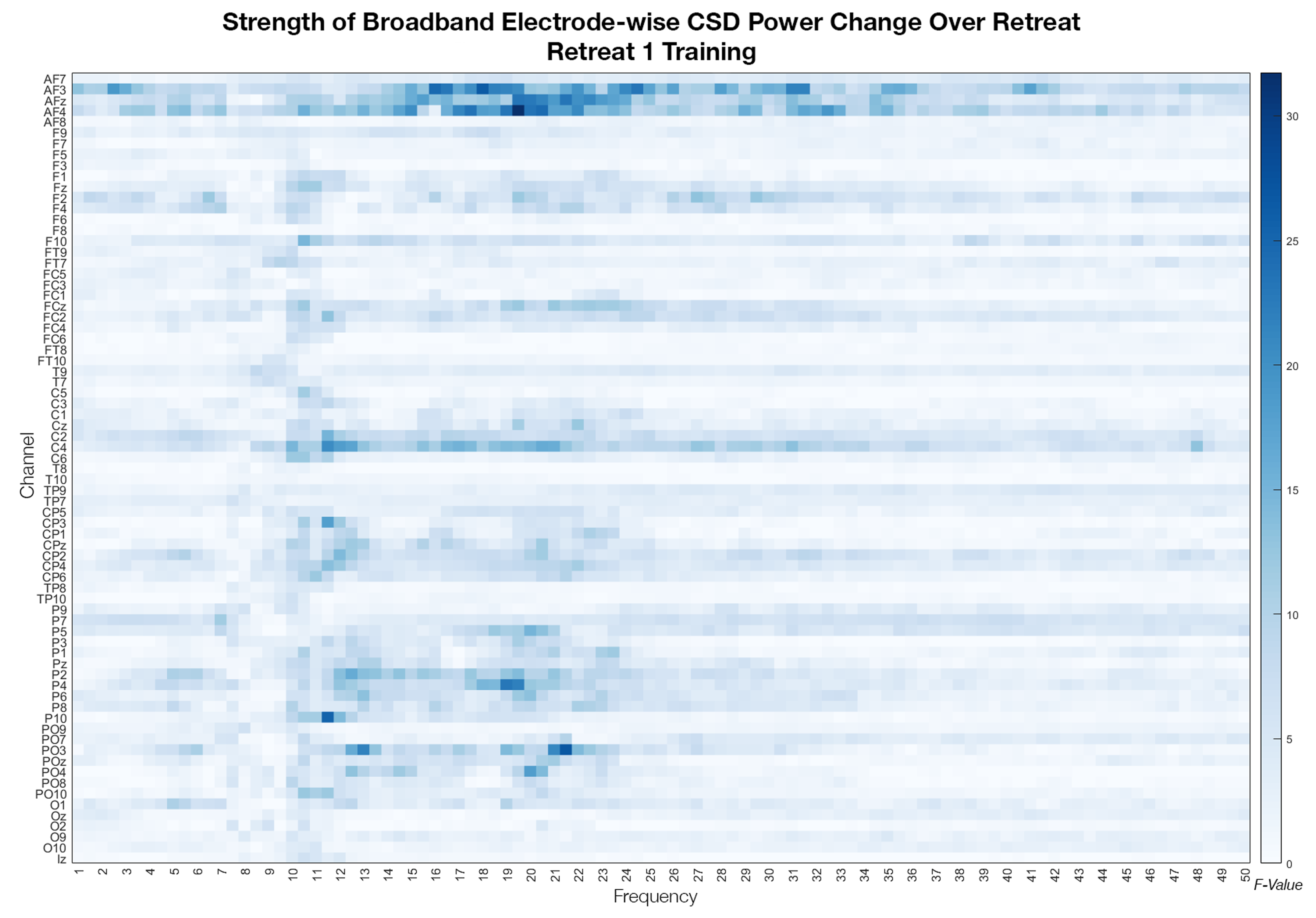

Supplement: Supplementary file 3 — (PNG 965 kb) [file 12671_2022_1974_Fig6_ESM.png]

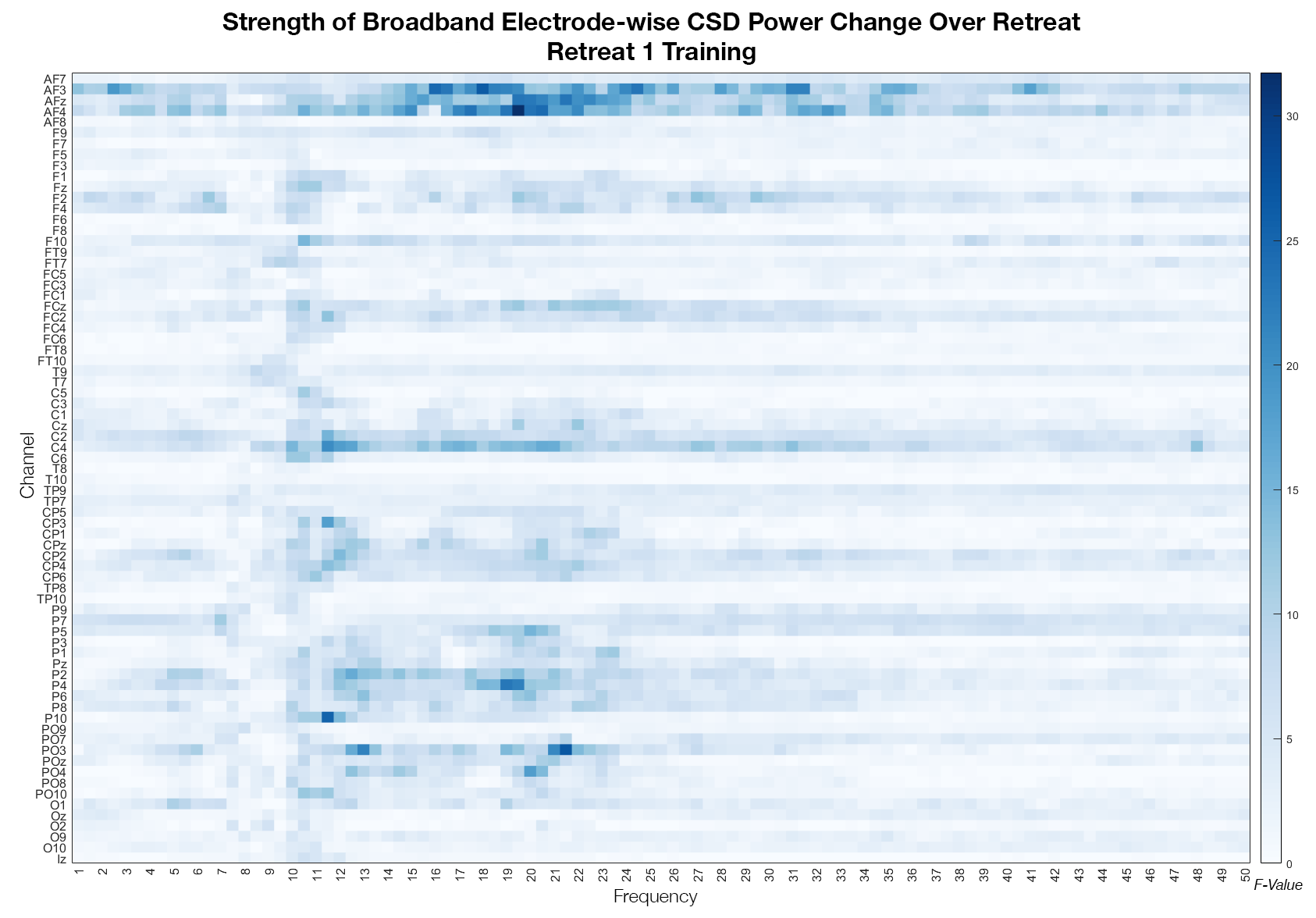

Supplement: Supplementary file 4 — High resolution image (TIF 5830 kb) [file 12671_2022_1974_MOESM2_ESM.tif]

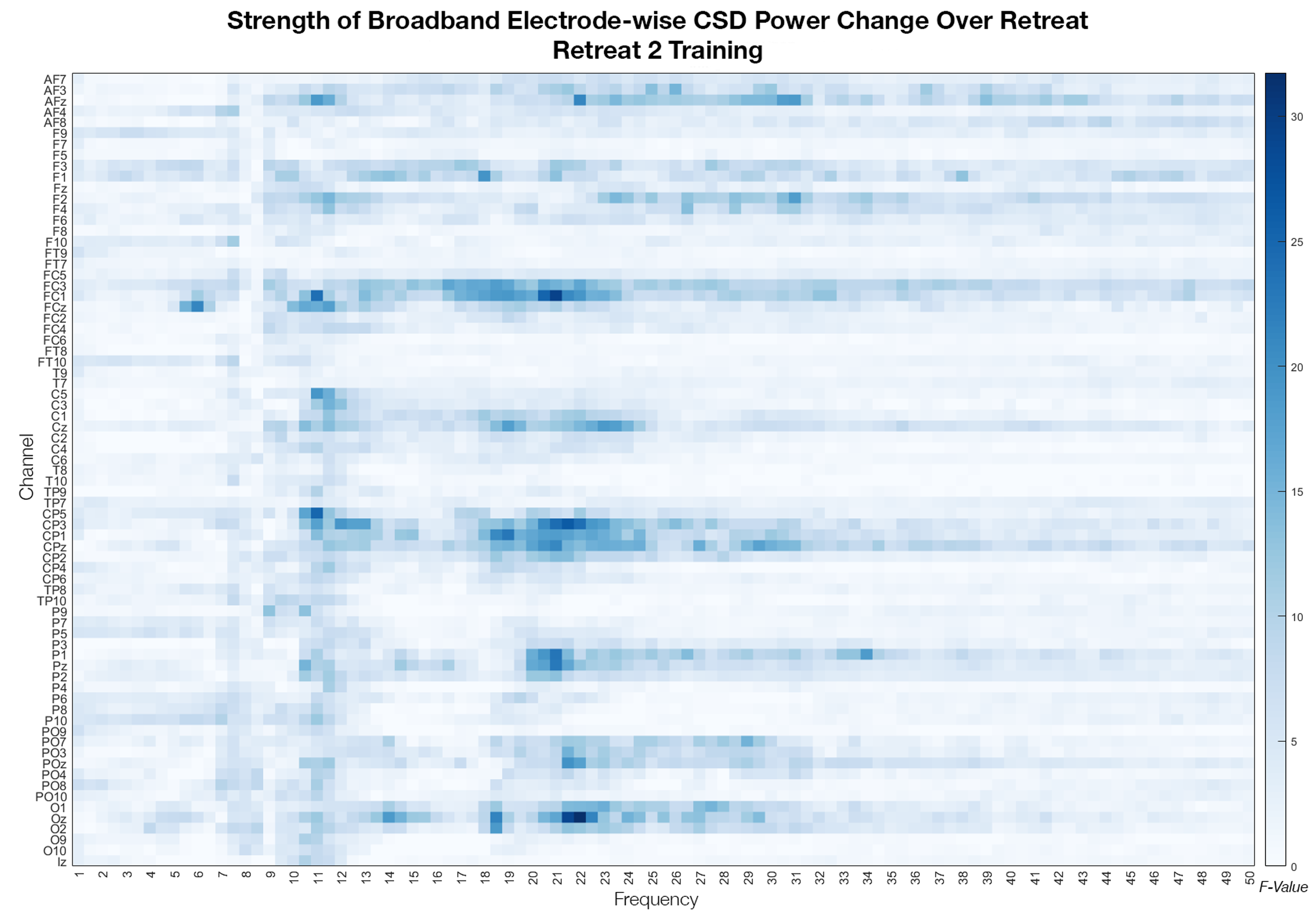

Supplement: Supplementary file 5 — (PNG 979 kb) [file 12671_2022_1974_Fig7_ESM.png]

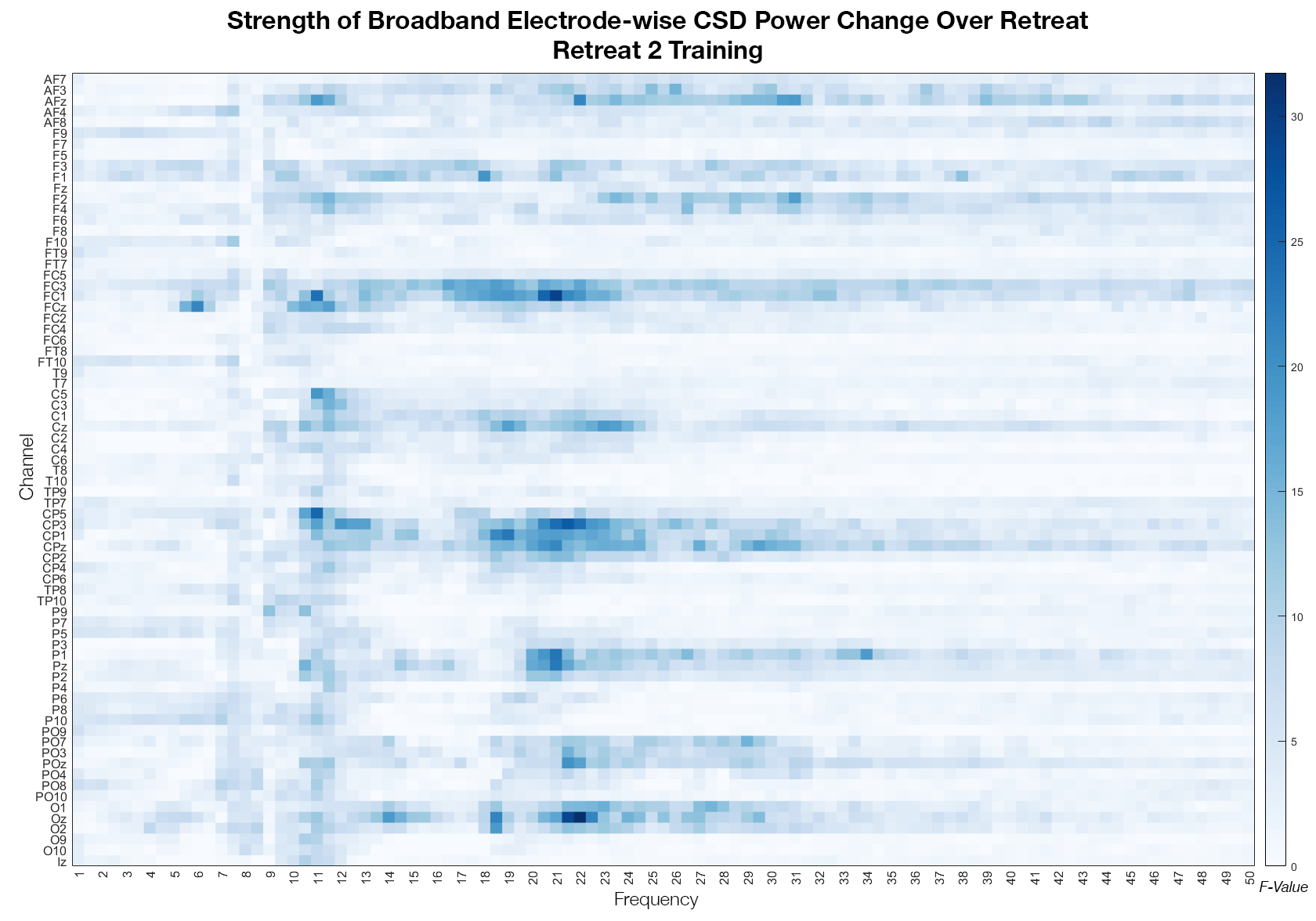

Supplement: Supplementary file 6 — High resolution image (TIF 5810 kb) [file 12671_2022_1974_MOESM3_ESM.tif]

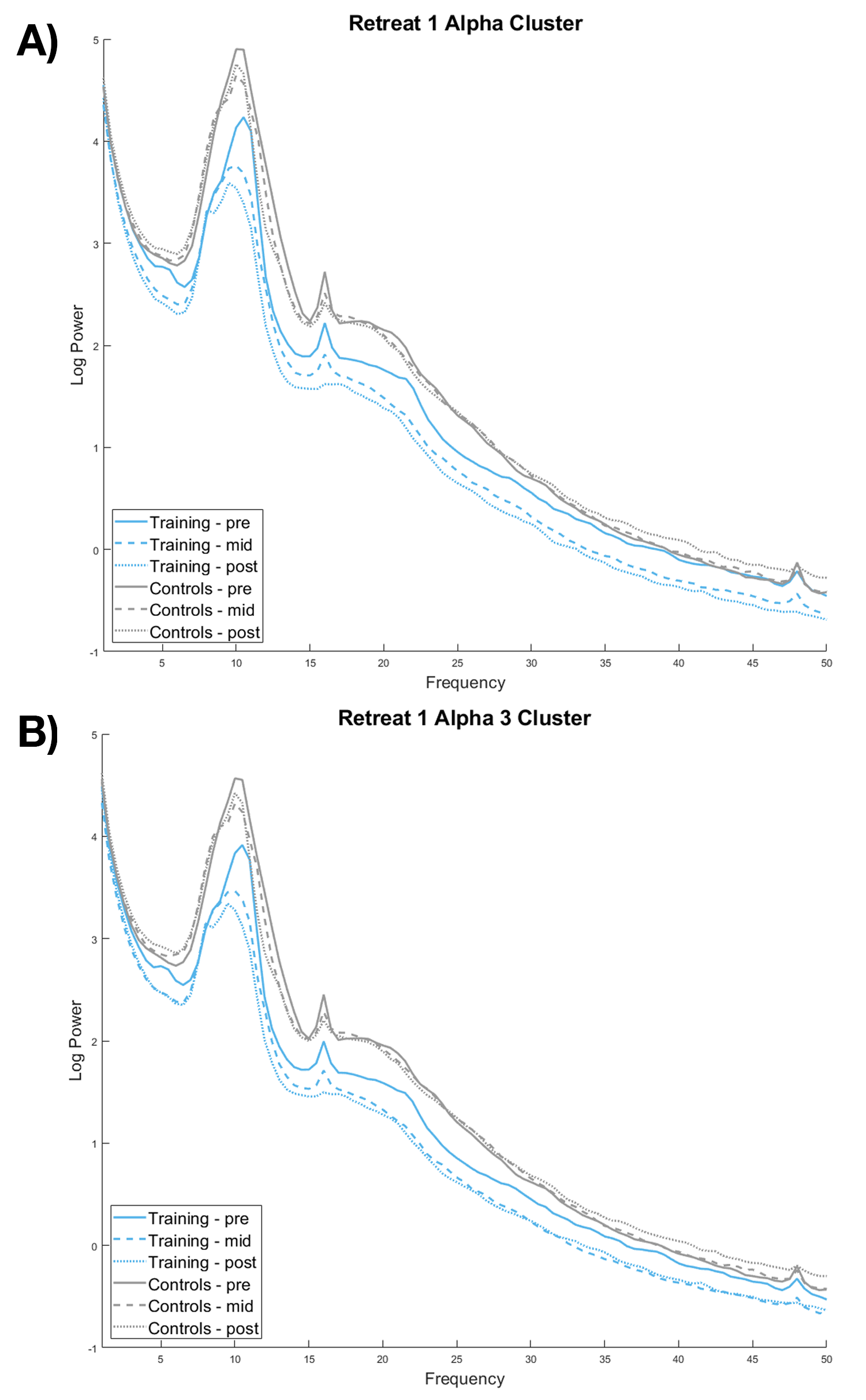

Supplement: Supplementary file 7 — (PNG 477 kb) [file 12671_2022_1974_Fig8_ESM.png]

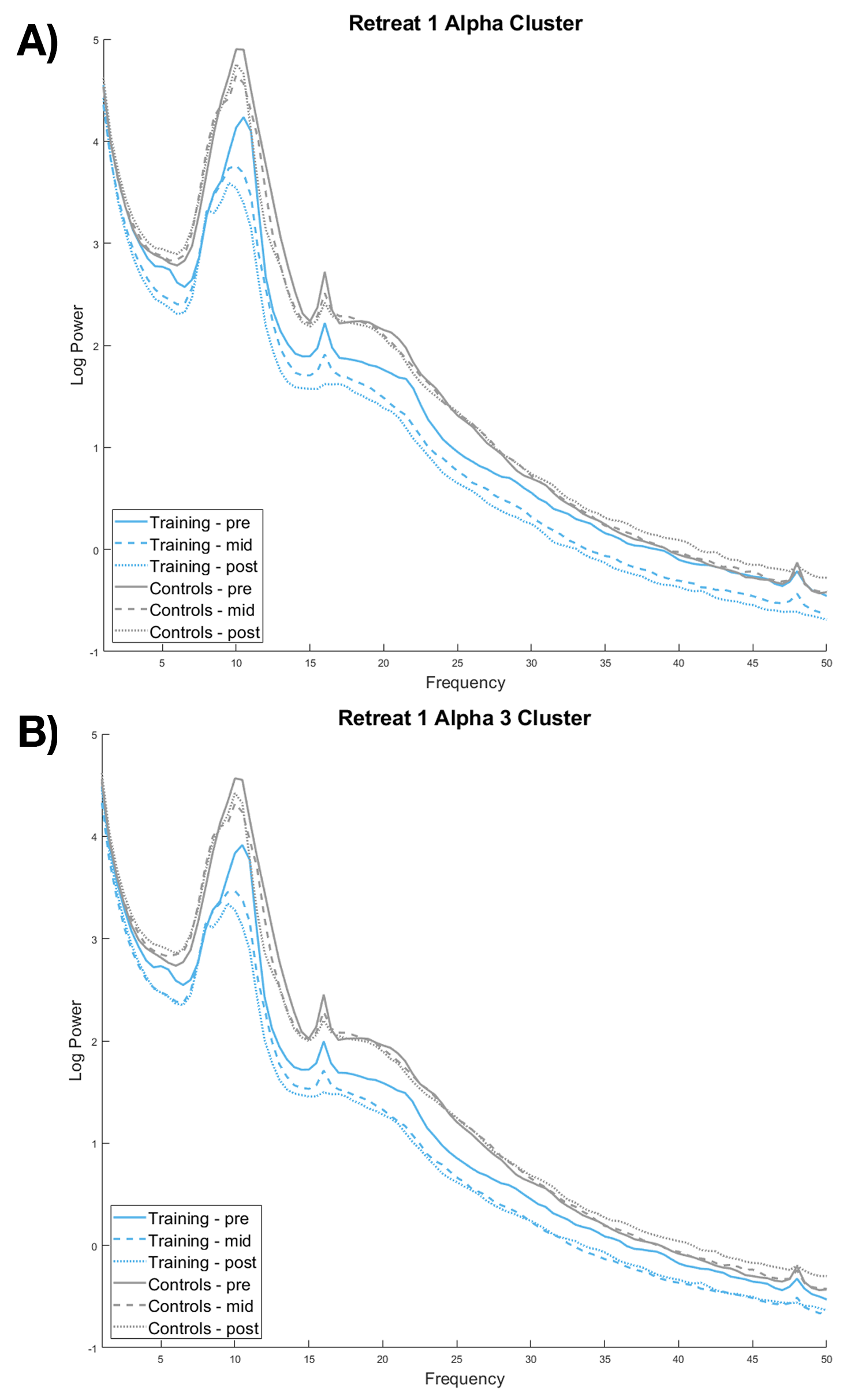

Supplement: Supplementary file 8 — High resolution image (TIF 10685 kb) [file 12671_2022_1974_MOESM4_ESM.tif]

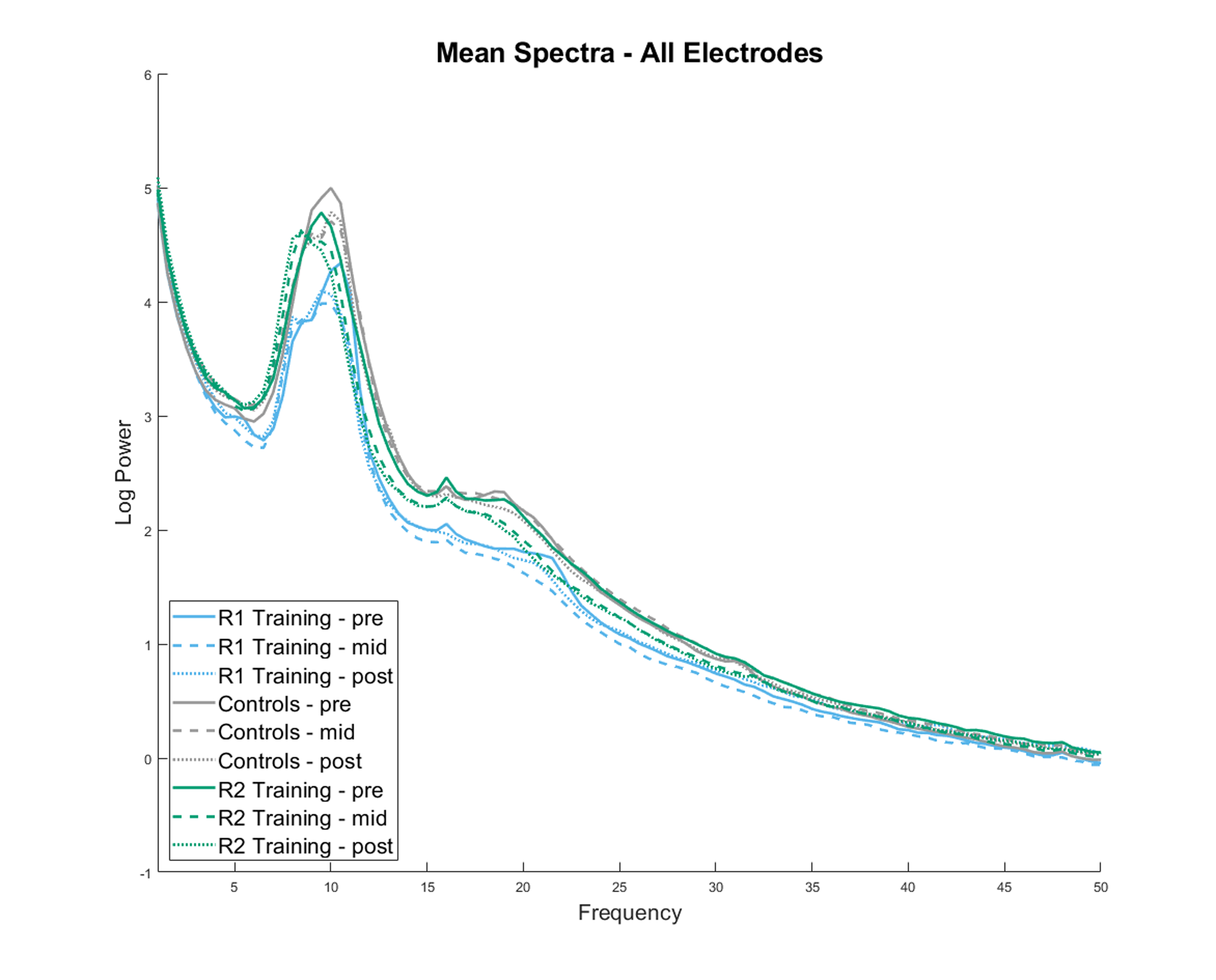

Supplement: Supplementary file 9 — (PNG 895 kb) [file 12671_2022_1974_Fig9_ESM.png]

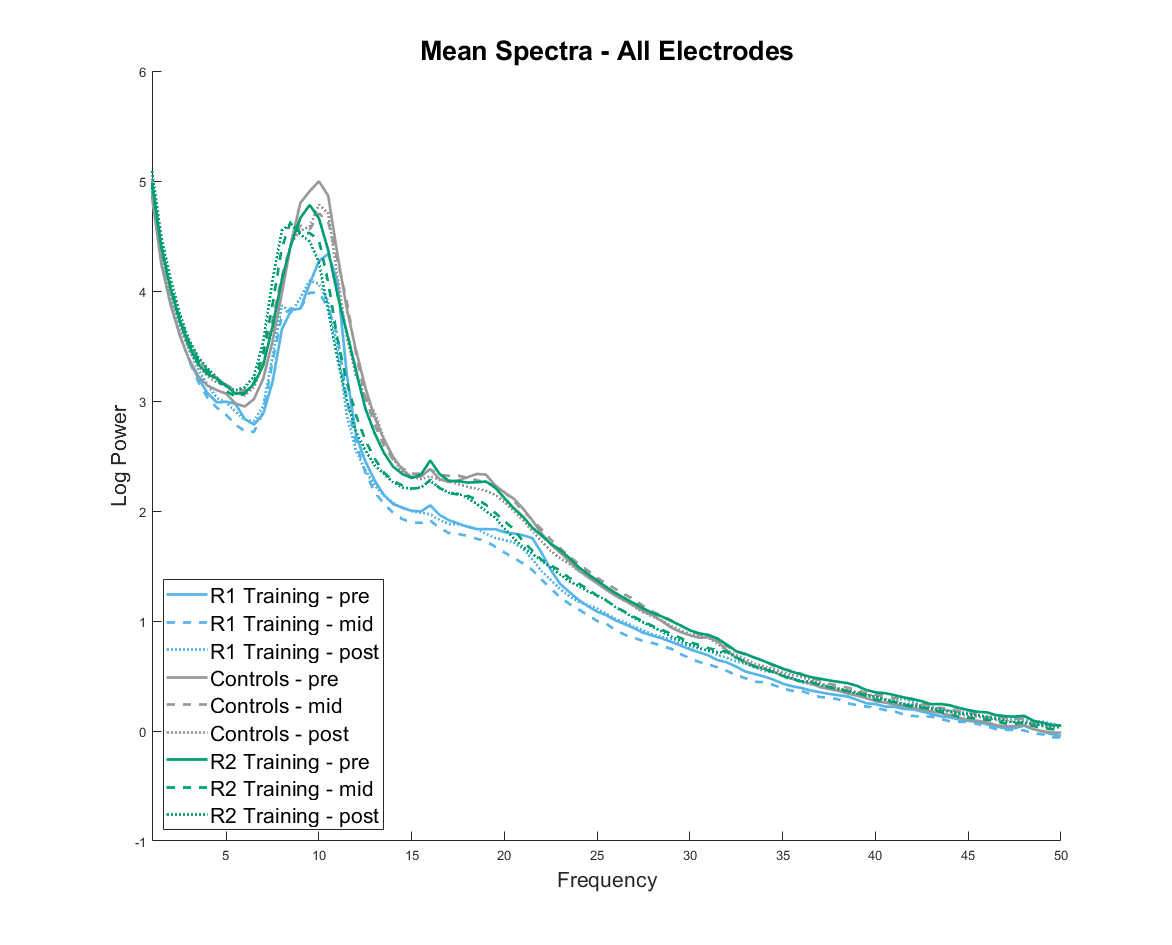

Supplement: Supplementary file 10 — High resolution image (TIF 152 kb) [file 12671_2022_1974_MOESM5_ESM.tif]
