# Supplementary material for: Shifting Baselines: Longitudinal Reductions in EEG Beta Band Power Characterize Resting Brain Activity with Intensive Meditation
Source: Mindfulness (N Y). 2022 Sep 20;13(10):2488–506. doi: 10.1007/s12671-022-01974-9 (PMC9568471; doi:10.1007/s12671-022-01974-9)
Supplement: Supplementary file 11 — (DOCX 18.8 kb) [file 12671_2022_1974_MOESM6_ESM.docx]

**Supplementary Information**

**Verification of Spectral Findings Using Canonical Frequency Bands**

We conducted an additional set of analysis to verify that the observed changes in spectral power were not merely a feature of changes in IAF-based band definitions over the course of retreat. We repeated all primary analyses using canonical fixed frequency bands, following identical data processing and analysis procedures as outlined in the Methods, with the exception that canonical fixed frequency bands rather than IAF-based frequency bands were used for power spectral estimation (see Table 1 of the manuscript for canonical band definitions). Cluster based permutation tests were run on the five frequency bands (delta, theta, alpha, beta, gamma) to identify clusters of change, and mixed models were used to assess group differences in change over time in clusters demonstrating significant change.

Using canonical frequency bands, we identified change in two bands in Retreat 1 training participants: in alpha, *cluster statistic* = 112.43, *p* = .009; and in beta, *cluster statistic* = 133.16, *p* = .007. In Retreat 2 training participants we identified one significant cluster of change in beta power, *cluster statistic* = 266.78, *p* < .001. One cluster in alpha was also identified in Retreat 2, but fell short of statistical significance, *cluster statistic* = 37.81, *p* = .052. No significant clusters of change were identified in Retreat 1 waitlist controls. Supplementary Figure 1 depicts the identified clusters of change using canonical frequency bands.

Mixed models were used to compare changes in mean power between groups from identified clusters. As with the IAF-based clusters, clusters identified in Retreat 1 and Retreat 2 active training participants were applied to Retreat 1 waitlist controls to provide a basis for between group comparisons. Model specification was identical to those used in the primary analysis.

For the Retreat 1 alpha cluster, there were significant main effects of assessment, *F*(2, 100) = 5.51, *p* = .005, and status *F*(1, 50) = 10.61, *p* = .002, but the interaction between assessment and status was not significant, *F*(2, 100) = 2.55, *p* = .083, and therefore no follow up tests were conducted.

In the cluster identified in the beta band, there were significant main effects of assessment, *F*(2, 100) = 8.04, *p* < .001, and status *F*(1, 50) = 15.78, *p* < .001, as well as a significant interaction between assessment and status *F*(2, 100) = 4.86, *p* = .010. Tests of simple effects demonstrated a significant effect of assessment in training participants, *F*(2, 100) = 12.14, *p* < .001, but not in controls, *F*(2, 100) = 0.31, *p* = .733. Follow-up comparisons indicated that active training participants had slightly lower cluster mean beta band power at the pre-retreat assessment than did waitlist controls (b = -0.47, *SE* = 0.16, *p* = .006, 95% CI [-0.79, -0.13]). Additional comparisons indicated that training participants decreased significantly from pre- to mid-retreat (b = -0.22, *SE* = 0.07, *p* = .001, 95% CI [-0.35, -0.09]), and from pre- to post-retreat (b = -0.33, *SE* = 0.07, *p* < .001, 95% CI [-0.46, -0.19]), but not from mid- to post-retreat (b = -0.11, *SE* = 0.07, *p* = .122, 95% CI [-0.24, 0.03]). Consistent with these patterns, training participants had significantly lower cluster mean beta band power than waitlist controls at the mid-retreat (b = -0.64, *SE* = 0.16, *p* < .001, 95% CI [-0.97, -0.31]) and post-retreat (b = -0.76, *SE* = 0.16, *p* < .001, 95% CI [-1.09, -0.43]) assessments.

In Retreat 2, the mixed model comparing active training participants to their prior status as controls showed significant main effects of assessment, *F*(2, 124) = 4.49, *p* = .013, and status, *F*(1, 127) = 16.89, *p* < .001, in the identified beta cluster, as well as a significant interaction between assessment and status, *F*(2, 124) = 5.57, *p* = .005. Tests of simple effects revealed that Retreat 2 participants significantly changed in beta power over assessments when they were active retreat participants, *F*(2, 124) = 9.83, *p* < .001, but not while they were waitlist controls, *F*(2, 124) = 0.04, *p* = .961. Follow-up comparisons indicated that these participants showed no difference in power in the identified beta cluster at the beginning of Retreat 1 versus the beginning of Retreat 2 (b = 0.01, *SE* = 0.07, *p* = .870, 95% CI [-0.12, 0.15]). Further comparisons indicated that these participants decreased significantly in cluster mean beta band power from pre- to mid-retreat during Retreat 2 (b = -0.24, *SE* = 0.07, *p* < .001, 95% CI [-0.38, -0.11]), and from pre- to post-retreat (b = -0.27, *SE* = 0.07, *p* < .001, 95% CI [-0.41, -0.14]), but not from mid- to post-retreat (b = -0.03, *SE* = 0.07, *p* = .625, 95% CI [-0.17, 0.10]). Consistent with this, Retreat 2 training participants had significantly lower cluster mean beta band power at the mid-retreat (b = -0.24, *SE* = 0.07, *p* < .001, 95% CI [-0.37, -0.10]) and post-retreat (b = -0.28, *SE* = 0.07, *p* < .001, 95% CI [-0.42, -0.15]) assessments than they did as waitlist controls.

The analysis using canonical frequency bands identified similar clusters that demonstrated a similar pattern of change as when IAF-based frequency bands are employed. Furthermore, we checked for correlations between IAF and cluster mean beta power in the clusters identified using IAF-based band definitions, collapsing across all training participants in both retreats. These analyses did not indicate any relationship between IAF and cluster mean beta band power at any assessment, nor between changes in these measures, all *p*s > .314. Together, these analyses suggest that our findings are not an artifact of shifts in IAF-based band definitions resulting from changes in IAF.

**Visualizing the Influence of Frequency Bands**

Our cluster analyses identified significant changes in beta as well as high alpha frequency bands. A natural question is whether these clusters represent distinct phenomena or are an artifact of the use of defined frequency bands, which could have artificially segmented a single continuous series of frequencies into two separate bands (alpha and beta). To address this issue, we conducted an additional cluster analysis on continuous frequencies rather than pre-defined frequency bands. Specifically, we submitted CSD power at 0.5 Hz intervals from 1 Hz to 50 Hz to the permutation procedure. This approach did not identify any clusters that reached statistical significance in Retreat 1 training participants. In Retreat 2, one cluster reached significance, *cluster statistic* = 5997.7, *p* = .003. This cluster encompassed frequencies from 9 Hz to 31.5 Hz. No clusters in waitlist controls approached significance.

Electrode-wise *F*-values from these analyses are shown in Supplementary Figures 2 and 3, which provide a visualization of patterns of change independent of pre-defined frequency bands. These heatmaps demonstrate broadband patterns of change, concentrated around frequencies canonically associated with alpha and beta bands. Although these visualizations do not conclusively answer whether the observed changes in high alpha and beta are a single or separate phenomena, they do illustrate some distinction between the two ranges. Additionally, they suggest that the changes observed in the band-based cluster analysis were not purely an artifact of band definition, as maximal *F*-values seem to be concentrated in the frequency ranges found to change in the band-based analyses.

**Additional Visualizations of Cluster Spectra**

Supplementary Figure 4 displays the power spectra from 1 to 50 Hz for the electrodes comprising the significant clusters identified in alpha and alpha 3 in Retreat 1, and Supplementary Figure 5 displays the spectra averaged across all electrodes for each group. These plots are intended to offer a more in-depth depiction of spectral dynamics for interested readers. Note that the peak at 16 Hz represents a known artifact resulting from the excitation frequency of electrodermal measurement in the Biosemi system used for recording, and appears particularly pronounced in the subsets of electrodes comprising the clusters identified in alpha and alpha 3 (Supplementary Figure 4). As the alpha band had a mean upper limit of 11.80 (see Table 2 in main manuscript), 16 Hz was not included in this band definition and we can be confident that the artifact peak does not contribute to these findings. Additionally, the electrode-by-frequency heatmaps displayed in Supplementary Figures 2 and 3 do not indicate exaggerated power change at 16 Hz.

**Supplementary Figures**

Supplementary Figure 1. Identified clusters of CSD power change using canonical fixed frequency bands in Retreat 1 (*n* = 25) and Retreat 2 (*n* = 26) training participants. No clusters were identified in waitlist controls.

Supplementary Figure 2. Electrode-wise F-values in Retreat 1 training participants (n = 25) obtained from a broadband non-parametric permutation-based cluster analysis. F-values indicate the strength of CSD power change for each electrode across retreat.

Supplementary Figure 3. Electrode-wise F-values in Retreat 2 training participants (n = 26) obtained from a broadband non-parametric permutation-based cluster analysis. F-values indicate the strength of CSD power change for each electrode across retreat.

Supplementary Figure 4. Average power spectra for electrodes comprising the significant clusters of change identified in alpha (panel A) and alpha 3 (panel B), respectively, during Retreat 1. Clusters were identified in Retreat 1 training participants (*n* = 25) and applied to waitlist controls (*n* = 27) for comparison. Note that the peak at 16Hz is a known artifact due to the excitation frequency of the electrodermal measurement in the Biosemi system used for recording and does not reflect neural activity. This frequency did not contribute to observed effects as it falls above the upper limit of the IAF-defined alpha range in which this cluster was identified.

Supplementary Figure 5. Mean power spectra across all 73 electrodes in Retreat 1 training (n = 25) and waitlist control (n = 27) participants, as well as Retreat 2 training participants (n = 26). Note that the peak at 16Hz is a known artifact due to the excitation frequency of the electrodermal measurement in the Biosemi system used for recording and does not reflect neural activity.
